# Peptide stereocomplex cross-links for polymer hydrogels†

**DOI:** 10.1039/d5sc00251f

**Published:** 2025-06-02

**Authors:** Israt Jahan Duti, Jonathan Paul, Keelin S. Reilly, Darren R. Miller, Diane A. Dickie, Rachel A. Letteri

**Affiliations:** a Department of Chemical Engineering, University of Virginia Charlottesville VA 22903 USA rl2qm@virginia.edu; b Department of Biomedical Engineering, University of Virginia Charlottesville VA 22904 USA; c Department of Chemistry, University of Virginia Charlottesville VA 22904 USA

## Abstract

Stereocomplexation, or stereochemistry-directed complexation between complementary stereoregular macromolecules such as polymers and peptides, brings about remarkable changes in the thermomechanical properties and stability of materials. Peptide stereocomplexes tie together these merits of stereocomplexation with the vast compositional space and biological function of peptides, and therefore are compelling building blocks of highly tunable, functional materials. In this work, we introduce peptide stereocomplexes as cross-links in polymer hydrogels. Attaching either l- or d-peptides to 4-arm PEG furnishes conjugates that are soluble in aqueous buffer, while their 1 : 1 blends form hydrogels at or above 7.5% (w/v). Increasing conjugate concentration increases both shear storage modulus (*G*′) and the intensity of the characteristic β-sheet infrared absorption at 1630 cm^−1^, highlighting the importance of peptide secondary structure for gelation. These gels, having peptide stereocomplexes as cross-links, strain stiffen up to nearly 50% strain, then soften at higher strains. Despite the crystalline nature of stereocomplexes, these gels display dynamic behavior: after application and removal of high strain, the gels recover partially, with 10–50% recovery of *G*′ after the first cycle and 50–70% in subsequent cycles. Moreover, the peptide stereocomplex cross-links imbue proteolytic stability, with nearly 80% of conjugates remaining intact after a 1 h incubation with Proteinase K, compared to just ∼40% of the l-conjugates. We anticipate that the material platform and combination of characterization methods presented here will readily extend to studying other peptides sequences, so as to leverage the full range of peptide design space and accelerate the development and implementation of peptide stereocomplexes to control hydrogel properties, function, and lifetime.

## Abstract 2

Peptide stereocomplexes cross-link polymers into hydrogels with enhanced mechanical properties and proteolytic stability relative to the individual constituent peptide–polymer conjugates.

## Graphical abstract

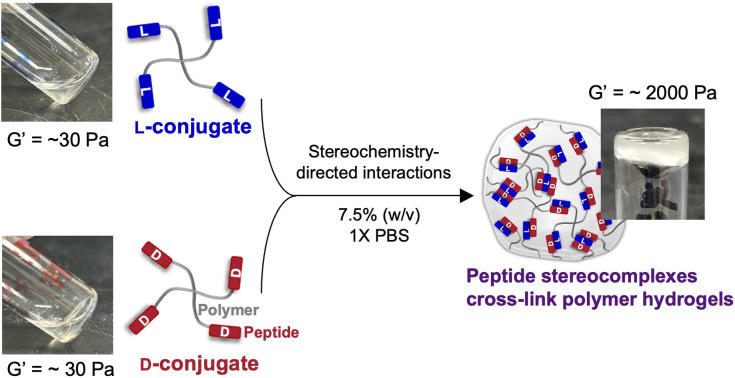

## Introduction

Stereochemistry-directed interactions between complementary stereoregular macromolecules, or stereocomplexation, is an impactful materials design tool, markedly modulating mechanics, morphology, crystallinity, and lifetime, among other properties.^1–23^ For instance, poly(l-lactic acid) (PLLA) and poly(d-lactic acid) (PDLA) melt at 170–180 °C, while their 1 : 1 stereocomplex blends melt nearly 50 °C higher at 220–230 °C.^4,5^ The half-life of PLLA heptamers in aqueous buffer at pH 7 and at 37 °C is 1 h, whereas the corresponding stereocomplexes have a half-life of 3.5 days.^12–14^ Additionally, stereocomplexes of isotactic and syndiotactic poly(methyl methacrylate) are semi-crystalline while individually these polymers are amorphous.^6–8^ The distinctive ability of stereocomplexes to alter material properties and the specificity of these interactions has also led to their use in organizing nanoparticles^8^ and polymers into supramolecular materials,^12–15,22,24–28^ including cross-linking polymers into networks.

The use of poly(lactic acid) (PLA) stereocomplexes to cross-link polymer hydrogels serves as an exciting precedent and highlights opportunities for stereocomplexation as a cross-linking mechanism.^12–15,22,24–28^ Blending solutions of dextran grafted with l-lactic acid oligomers and dextran grafted with d-lactic acid oligomers in a 1 : 1 ratio yields hydrogels, while the graft polymers individually remain soluble.^12–14,29^ Similarly, blends of PLLA- and PDLA-functionalized 8-arm polyethylene glycol (PEG), *i.e.*, PEG-(PLLA)_8_ and PEG-(PDLA)_8_, gel at lower concentrations than either copolymer alone.^26^ The Becker lab recently showed mixtures of PDLA and 4-arm PEG functionalized with PLLA, *i.e.*, PDLA and PEG-(PLLA)_4_, to form semi-crystalline hydrogel microparticles, whereas microparticles formed in absence of PDLA are amorphous.^15,22^ Yet, the compositional space and functionality of PLA and therefore PLA-based stereocomplexes is limited. Underscoring this point, they used creative synthetic strategies to introduce alkynes^15^ for added functionality onto the PDLA constituent of the hydrogel microparticles.

Peptides have a vast compositional design space, well suited for the design of tunable, highly functional cross-links.^30–35^ Like PLA, they have l- and d-isomers that stereocomplex into materials with distinct stiffnesses, stability, and morphology from either isomer alone. For example, the shear moduli of fibrous hydrogels formed from 1 : 1 l : d-mixtures of the β-sheet peptide ‘MAX1’ are four times higher than those from the individual l- and d-peptide.^16,17,36^ In another example, stereocomplexation of the l- and d-amyloid β peptides Aβ(16–22) produces a morphological transition from nanoscale fibers to micron-scale needle-like structures.^37^ We recently showed for the peptide KYFIL^38,39^ in 1× PBS and at pH 7.4, a similar stereocomplexation-directed change in morphology from fibers to plates, which lowers stiffness and even inhibits gelation, as the entangleable fibers transform into unentangleable plates.^20^ KYFIL stereocomplexation also imparts crystallinity, with l- and d-KYFIL individually being largely amorphous whereas their blends are crystalline.^20^ Moreover, KYFIL^20^ and other peptide stereocomplexes^19,36^ confer proteolytic stability, providing opportunities to control material lifetime. These reports attest the tunability of peptide stereocomplexes and their suitability as cross-links, yet, to our knowledge, they have not been employed in this capacity. We anticipate that the diverse sequences, hierarchical structures, and biological functions of peptides can be utilized to tune the molecular scale interactions of stereocomplexed cross-links and, by extension, the bulk properties of the resulting hydrogels.

In this manuscript, we introduce peptide stereocomplexes as cross-links for polymer networks. We selected KYFIL as the peptide sequence for this study, since despite the morphological transformation that prevents gelation of the unconjugated peptides, l- and d-KYFIL clearly undergo stereochemistry-directed assembly that suggests their promise as cross-links. We show that KYFIL stereocomplexation capably promotes gelation, as blending solutions of conjugates decorated with l-peptides and conjugates decorated with d-peptides yields hydrogels. Gelation is accompanied by β-sheet formation, as both increase with concentration. Despite the crystalline nature of stereocomplexes, these gels flow upon application of high strain and recover partially following application and removal of high strain. Relative to the l-conjugates alone, the stereocomplexed gels exhibit enhanced proteolytic stability, attesting to the utility of peptide stereocomplexes as cross-links, particularly when controllable lifetimes in biological environments are desired.

## Results and discussion

### Synthesis of peptide–polymer conjugates

**Fig. 1:**
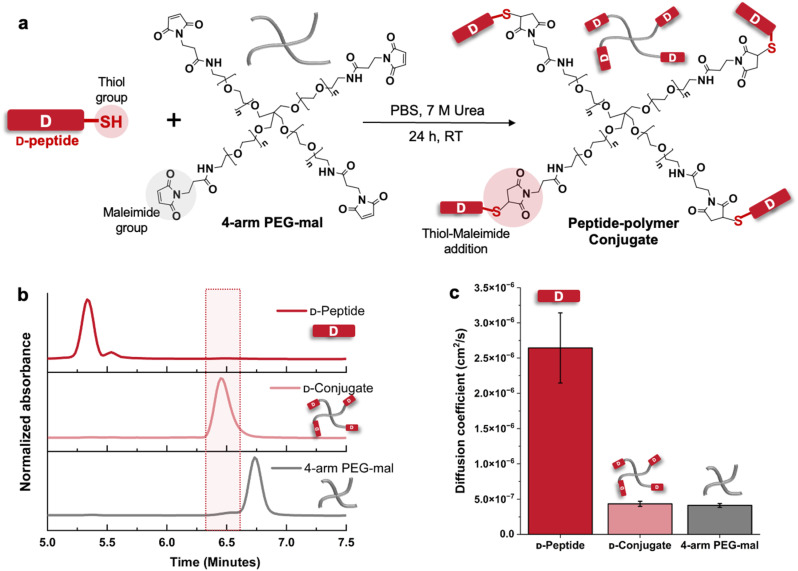
Conjugation of KYFILC peptides with 4-arm PEG20k-maleimide, showing preparation of d-KYFILC conjugates here, whereas analogous data for l-KYFILC are provided in Fig. S11 and S19.† (a) Schematic of the reaction between the thiol group on the peptide and the maleimide groups on the polymer to form conjugates, and (b) HPLC traces of d-KYFILC, 4-arm PEG-mal, and the resulting conjugates. The distinct elution time of the conjugate relative to those of peptide and polymer supports conjugation. (c) Diffusion coefficients of d-KYFILC, 4-arm PEG-mal, and d-conjugate obtained from diffusion ordered spectroscopy in D_2_O, with the decrease of diffusion coefficients of the peptide-associated peaks in the conjugate to match those of the polymer further indicating the attachment of peptide to polymer.

While we could conceivably use any synthetic star-shaped or branched polymer for studying peptide stereocomplex-mediated gelation, we selected 4-arm PEG since it is a well-defined and extensively studied hydrogel material,^40–42^ allowing us to focus on the role of the peptide stereocomplex cross-links. To attach the peptides to PEG, we selected a thiol–maleimide reaction^43,44^ due to its efficiency under mild conditions (Fig. 1a). Therefore, we prepared cysteine-terminated l- and d-KYFIL, or l-KYFILC and d-KYFILC (Fig. S1–S8†), for conjugation to maleimide-functionalized 4-arm PEG. Similarly as we reported previously for KYFIL,^20^l- and d-KYFILC are soluble, random coils in water and form β-sheets and turbid suspensions in PBS at 3% (w/v).

Model reactions between KYFILC and linear (1-arm) PEG maleimide did not initially proceed in 1× PBS (Fig. S9a†). Guided by prior reports showing urea, a hydrogen bond disruptor, to promote conjugation of β-sheet peptides to polymers,^45–50^ we added 7 M urea to 1× PBS, which resulted in conjugation (Fig. S9b†). In the 4-arm conjugations, we used a slight excess of peptide (1.25 equivalents relative to polymer maleimides) to maximize reacted polymer chain ends. We note that KYFILC peptides are only partially soluble and form a white dispersion in the 7 M urea 1× PBS solution (Fig. S10†). As the reaction proceeds, the mixture clarifies, suggesting peptide conjugation to the soluble PEG. To remove urea, salts, and excess peptide after the reaction, we dialyzed the reaction mixture and lyophilized the conjugates, isolating them in ∼90–95% yield.

To confirm that the conjugation reaction and the subsequent dialysis proceeded as intended, we performed high performance liquid chromatography (HPLC), size exclusion chromatography (SEC), ^1^H nuclear magnetic resonance (NMR) spectroscopy, diffusion ordered spectroscopy (DOSY), and circular dichroism (CD) spectroscopy on the peptides, polymers, and conjugates. Both HPLC and SEC chromatograms of the isolated conjugates show no remaining peptide, indicating the removal of excess peptide during dialysis (Fig. 1b and S11–S13†). The distinct HPLC elution time of the conjugates (6.5 min) relative to those of the peptide (5.3 min) and polymer (6.75 min) supports conjugation. Since SEC showed just a small shift in elution time between the polymer and the conjugate, we verified the synthesis of larger conjugate structures with DOSY.^51^ We found the diffusivities of the peptide resonances in the conjugate spectra (4 × 10^−7^ cm^2^ s^−1^) to be similar to those of the polymers (4 × 10^−7^ cm^2^ s^−1^) and smaller than those of the unconjugated peptides (27 × 10^−7^ cm^2^ s^−1^) (Fig. 1c and S17–S19†), supporting peptide attachment to larger polymers. Further supporting the conjugation, comparing the ^1^H NMR spectra of 4-arm PEG-mal and the conjugates shows the disappearance of the maleimide peaks at 6.88 ppm upon conjugation, reflective of maleimide consumption (Fig. S14–S16†). To determine the percentage of PEG arms functionalized with peptide, we compared the relative integrations of the aromatic proton resonances of the peptides (6.5–7.5 ppm) in the conjugate spectra and the resonances of methylene protons situated beta to the maleimides on the polymers (2.5 ppm). Depending on the particular peptide resonance we selected for analysis, we determined 75–85% arms of the PEG were functionalized. CD confirmed that the conjugated peptides retained their stereochemistry and random coil secondary structure in pure water (Fig. S20†).

In 1× PBS, while 1.5% (w/v) unconjugated KYFIL forms β-sheets, the conjugates are soluble and we see no evidence of β-sheet formation by infrared (IR) spectroscopy even at 15% (w/v) (Fig. S21–S22†), corresponding to a peptide equivalent concentration of ∼2% (w/v), which exceeds the peptide concentrations in the 1.5% (w/v) formulations of peptide alone. As noted in our previous report on unconjugated KYFIL,^20^ we used IR here for secondary structure determination rather than CD because the high peptide concentrations and the presence of salt that promote β-sheet formation also complicate CD spectra acquisition by saturating the detector. That the conjugates do not form β-sheets at similar peptide concentrations as the unconjugated peptides suggests that conjugation to polymer partially inhibits interactions between peptides. We see this decrease of interaction upon conjugation as a benefit for keeping the l- and d-conjugates soluble to ensure mixing of the two components while preparing stereocomplexed hydrogels.

### Hydrogel formation with peptide stereocomplex cross-links

**Fig. 2:**
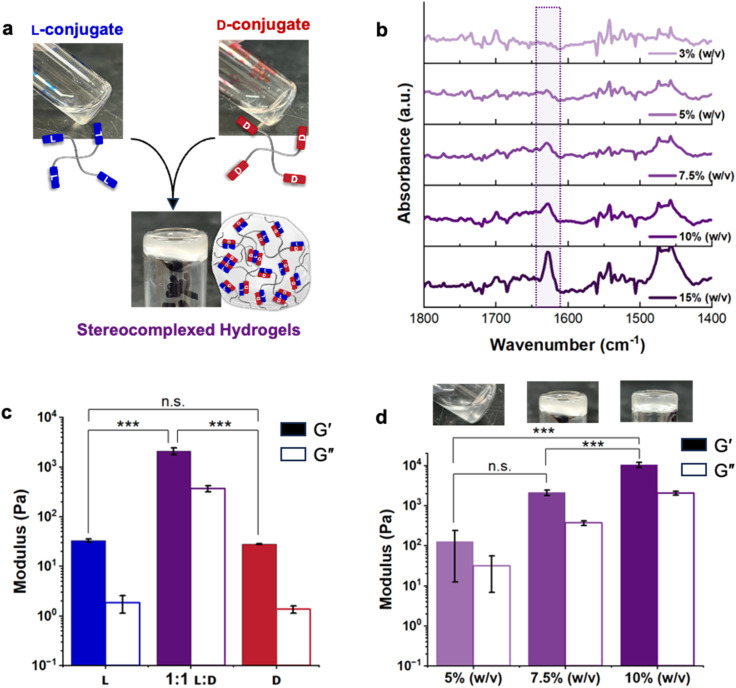
Gelation behavior and rheology of the stereocomplex-cross-linked conjugates in 1× PBS: (a) photographs showing hydrogel formation from a 1 : 1 mixture of l- and d-conjugates at 7.5% (w/v), while neither conjugate individually gels at the same concentration. (b) IR spectra of the 1 : 1 l : d mixtures of conjugates at 3, 5, 7.5, 10 and 15% (w/v) show β-sheet formation at concentrations of ≥7.5% (w/v), consistent with the critical gelation concentration we determined with the inversion tests. (c) The average shear moduli obtained from frequency sweeps show the 1 : 1 l : d conjugate mixture to have higher *G*′ than either individual conjugate at 7.5% (w/v), indicating stereocomplexation-promoted gelation. (d) The effect of concentration on hydrogel shear moduli, where we observe *G*′ to increase with concentration. Storage (*G*′) and loss (*G*′′) moduli are reported as the average modulus measured at 10 rad s^−1^ at 5% strain from 3 independently prepared samples, with error bars representing standard deviation.

We prepared l- and d-conjugates and their 1 : 1 volumetric mixtures at 3, 5, 7.5, and 10% (w/v) in 1× PBS and assessed critical gelation concentration by inversion tests after 24 h to allow the conjugates sufficient time to interact and promote gelation. Solutions of individual l- and d-conjugates remain soluble even at the highest concentrations. Yet, the stereocomplexed mixtures at 7.5 and 10% (w/v) form solid-like hydrogels, as indicated by the vial inversion test, suggesting stereocomplexation-promoted gelation with a critical gelation concentration of 7.5% (w/v) (Fig. 2a and S21†). The stereocomplexed mixtures at the highest concentration gel immediately, whereas gelation of the 7.5% (w/v) mixture requires longer than 1 h. While none of the individual conjugates show IR absorbance at ∼1630 cm^−1^ associated with β-sheet formation (Fig. S22†), the stereocomplexed hydrogels show significant absorbance at 1627–1635 cm^−1^ for all concentrations except 3 and 5% (w/v), which are below the critical gelation concentration. Moreover, the absorbances are more prominent at higher concentrations (Fig. 2b), signifying the relationship between secondary structure and hydrogel formation.

Oscillatory shear rheology, consistent with the inversion test images, shows viscous, liquid-like behavior of both l- and d-conjugates at all concentrations and viscoelastic, gel-like behavior for stereocomplexed hydrogels at concentrations ≥7.5% (w/v) (Fig. S23–28†). The average moduli, measured between 1–10 rad s^−1^ and at 1% strain from 3 independently prepared samples, of l-conjugates at 7.5% (w/v) are similar to those of d-conjugates, with storage modulus *G*′ = 12.9 ± 1.8 Pa and loss modulus *G*′′ = 4.1 ± 0.9 Pa for l-conjugates and *G*′ = 8.0 ± 0.4 Pa and *G*′′ = 0.4 ± 0.2 Pa for d-conjugates (Fig. 2c). While *G*′ approximately equals and even slightly exceeds *G*′′ for these individual conjugates, suggestive of weak hydrogel formation, the images in Fig. 2a and S21† showcase the liquid-like nature of these solutions. We suspect that the parallel plate geometry, selected for the stereocomplexed hydrogel samples with solid-like character, led to higher variability and artifacts in the liquid-like solutions of the individual conjugate controls and for stereocomplexed mixtures below the critical gelation concentration. In contrast, stereocomplexed hydrogels at the critical gelation concentration, 7.5% (w/v), exhibit significantly higher moduli (*G*′ = 1500 ± 300 Pa, *G*′′ = 350 ± 50 Pa) than those of the individual conjugates, demonstrating peptide stereocomplex cross-links to promote gelation (Fig. 2c and S28†). We found increasing conjugate concentration increases stiffness, with hydrogels at 10% (w/v) exhibiting *G*′ = 8000 ± 1800 Pa, *G*′′ = 2000 ± 250 Pa (Fig. 2d). These data are consistent with the findings from IR spectra in Fig. 2b showing β-sheet content to increase with concentration and underscore the role of secondary structure in gelation mediated by peptide stereocomplexes.

Comparing the hydrogels formed here to the materials formed upon stereocomplexation of unconjugated KYFIL that we reported previously,^20^ the changes in mechanical properties upon stereocomplexation differ distinctly. Stereocomplexation of unconjugated KYFIL peptides transforms fibrous hydrogels formed from l- and d-KYFIL individually into dispersions of crystalline micron-scale plates that cannot entangle or gel, and therefore stereocomplexation deteriorates mechanics. In contrast, here when KYFIL is attached to polymers, stereocomplexation leads to gelation and enhances mechanics.

### Dynamic behavior of the peptide stereocomplex cross-links

**Fig. 3:**
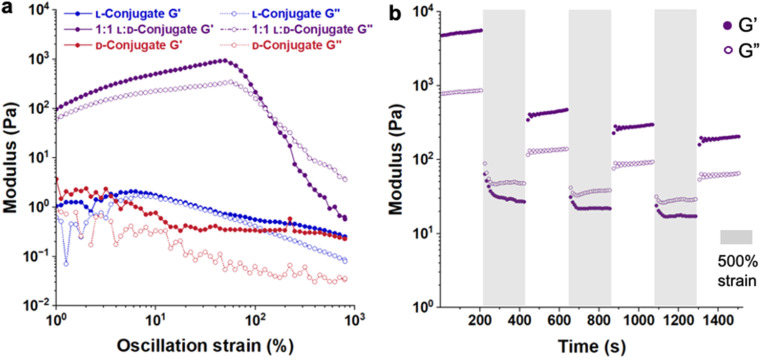
Dynamic behavior of stereocomplexed hydrogels in 1× PBS: (a) amplitude sweeps of KYFIL l-, d-, and 1 : 1 l : d-conjugates at 10% (w/v). Samples subjected to oscillatory shear using a 8 mm diameter parallel plate geometry with a 500 μm gap height at 1 rad s^−1^ and 25 °C, ramping logarithmically from 1% to 800% strain. The stereocomplexed hydrogels show strain-stiffening behavior with *G*′ increases slightly up to 50% strain. (b) Hydrogels were subjected to three cyclic applications of 500% strain for 200 s (grey area), followed by 5% strain for 200 s (white area). Hydrogels recover ∼10% after the first cycle, and for the remaining cycles the recoveries were ∼60%.

While stereocomplexes are crystalline in nature and KYFIL stereocomplexes are no exception,^20^ they are still non-covalent complexes, and therefore we sought to investigate the extent to which KYFIL cross-links imparts dynamic character to polymer hydrogels. First, to determine the strain required to induce a transition from elastic gel-like behavior to viscous liquid-like behavior, we performed a strain sweep (1–800%) at 1 rad s^−1^ on both 7.5% (w/v) and 10% (w/v) gels. *G*′ increases slightly up to 50% strain (Fig. 3a, S24 and 25†), and at higher strains drops precipitously and below *G*′′. From 3 replicate measurements for each of the two conjugate concentrations, we observed a crossover (*G*′ < *G*′′) at ∼300% strain. It is interesting to note that the stereocomplexed hydrogels strain-stiffen, whereas the liquid-like individual conjugates shear thin. Such strain-stiffening behavior is resemblant of dynamic covalent networks,^52,53^ spider silk,^54,55^ and biological tissue.^52,56^

To gauge the recovery of the hydrogels after exposure to high strain, next we applied and removed high strain in a cyclic manner at 1 rad s^−1^ to hydrogels prepared from 1 : 1 l : d conjugate mixtures. To exceed the crossover point (∼300% strain) and render the gels liquid-like, we applied 500% strain for 200 s, followed by low strain (5%) for 200 s, and repeated these steps three times. With each application of high strain, *G*′ drops below *G*′′ and upon removal of the strain the gels regain their viscoelastic gel-like character. However, *G*′ does not fully recover. The 7.5% (w/v) gels recovered ∼10–50% after the first cycle and ∼50–60% after the next two cycles (Fig. 3, S29 and S31†). Despite the higher *G*′, the 10% (w/v) gels behaved similarly, with ∼10–50% recovery after the first cycle and ∼60–70% recovery after the successive cycles (Fig. S30 and S31†).

When considering explanations for the incomplete recovery of these stereocomplexed hydrogels following high strain, the crystalline nature of these complexes came to mind. When not conjugated to polymers, l- and d-KYFIL are amorphous, whereas their stereocomplex blends are crystalline.^20^ We suspect that this crystalline character is retained in these hydrogels, as evidenced by characteristic β-sheet peaks in the IR spectra, and small but reproducibly distinct features in the X-ray diffraction (XRD) patterns of the lyophilized stereocomplexed hydrogels (Fig. S32†). Yet, similarly as observed for PEG hydrogels cross-linked with polylactide stereocomplexes,^28^ the crystallinity of PEG upon lyophilization dominates the XRD patterns and obscures features related to changes due to the stereocomplexes. Nevertheless, in two independently prepared sample sets, we observe an increase in the peak intensity at approximately 2*θ* = 27° for the stereocomplexed hydrogels relative to the patterns of either individual conjugate. Therefore it is certainly plausible that the crystalline nature the KYFIL stereocomplexes may be limiting the dynamic nature of these hydrogels. Going forward, it will be important to understand how peptide molecular features (*e.g.*, sequence, length, hydrophobicity, charge *etc.*) impact the resulting balance between l- and d-peptide crystallization, helpful for binding and cross-linking, and dynamic character, helpful for injectability and printing in biological application.

### Proteolytic stability of stereocomplexed hydrogels

**Fig. 4:**
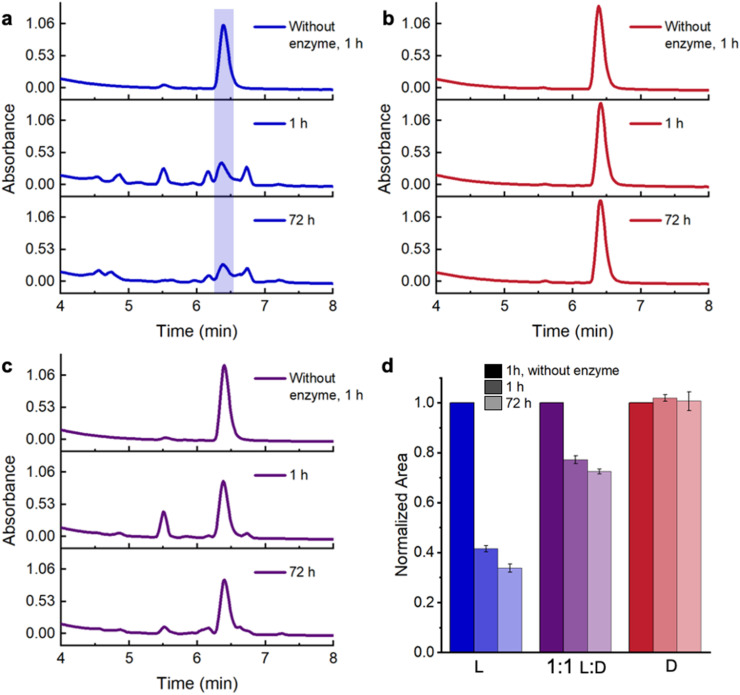
Proteolytic stability of hydrogels at 7.5% (w/v) in the presence of 0.1 mg per mL Proteinase K. HPLC chromatograms of (a) l-conjugates, (b) d-conjugates and (c) 1 : 1 l : d-hydrogels after 1 and 72 h. The appearance of new peaks following incubation with protease indicates degradation of l-conjugates, whereas we observe no degradation of d-conjugates and little evidence of degradation in the 1 : 1 l : d-hydrogels. (d) Fraction of intact conjugates remaining after 1 and 72 h incubation with Proteinase K as gauged by the normalized HPLC peak area, showing 1 : 1 l : d-KYFIL hydrogels to remain stable, with >70% KYFIL remaining intact after 72 h. The error bars represent standard deviation, from *n* = 3 samples.

Given the potential of peptide stereocomplexes to prolong hydrogel lifetime due to enhanced proteolytic stability, we were interested to learn whether the KYFIL stereocomplex cross-links boost the proteolytic stability of polymeric hydrogels. In our prior study with unconjugated KYFIL stereocomplexes, we incubated l-, 1 : 1 l : d-, and d-KYFIL in Proteinase K, an enzyme that is predicted to cleave l-KYFIL between the Y–F, F–I, and I–L residues.^57^ We found more than 90% intact peptide in the 1 : 1 l : d-peptide mixtures after incubation with Proteinase K for 72 h, while only ∼35% l-KYFIL remained intact.^20^ Similar to our previous study, we incubated l-, 1 : 1 l : d-, and d-conjugates, as well as 4-arm PEG20k maleimide at 7.5% (w/v) in the presence and absence of Proteinase K for 72 h. To maximize the distribution of enzyme throughout the hydrogels, we added it during gel formation. At each time point, we added dimethyl sulfoxide (DMSO) to each sample to solubilize the conjugates and deactivate Proteinase K before acquiring HPLC traces to determine the percentage of intact conjugate. We observed no degradation products absorbing at 214 nm from polymers without attached peptides in the presence of Proteinase K (Fig. S33 and S34†), allowing us to focus these measurements on the proteolytic stability of the peptide stereocomplexes in hydrogels. In the absence of Proteinase K, l-, 1 : 1 l : d-, and d-conjugates elute at 6.4 min and show no significant degradation after 72 h (Fig. 4a–c and S35–S37†). In the presence of Proteinase K, d-conjugates remained intact as expected throughout the duration of the experiment (Fig. 4b, d and S36†). However, only ∼41% of l-conjugates are intact after 1 h, while new peaks appear in the chromatograms at 4.5–6.3 min and ∼6.75 min, indicative of degradation (Fig. 4a, d and S35†). The degradation slows after 1 h, with ∼33% l-conjugate remaining after 72 h. In contrast, 77% and 72% of the peptide in the stereocomplexed hydrogels remained intact after 1 and 72 h respectively, with new peaks attributed to degradation products at ∼5–6.3 min and ∼6.75 min (Fig. 4cd and S37†). If stereocomplexation were not to shield the l-peptides from degradation, we would expect to observe 70% intact peptide after 1 h (50% is non-degradable d-conjugate +20% from the ∼40% of the l-conjugate that remains intact in the individual conjugate solutions) degradable of the conjugate is non-degradable d40% of the l-peptide is 20%); yet we observe slightly higher, nearly 80% intact conjugate after 1 h. These findings resemble our prior observations of unconjugated KYFIL peptides,^20^ and while the stability enhancement is not as large in the hydrated polymer networks, point to the ability of peptide stereocomplex cross-links to prolong the lifetime of biomaterials.

## Conclusions

Here we introduce stereochemistry-directed interactions of peptides, or peptide stereocomplexation, as a cross-linking mechanism for polymeric hydrogel formation that controls material mechanics and stability. Using KYFIL peptides conjugated to 4-arm star PEG, we show stereocomplexes of l- and d-peptides are sufficiently strong and specific to cross-link PEG into hydrogels. In contrast to the unconjugated peptides, where stereocomplexation of l- and d-KYFIL changes the morphology in a way that hinders gelation, blending solutions of 4-arm star l-conjugate and d-conjugate in a 1 : 1 ratio in PBS capably promotes gelation, as evident from visual observations and rheological measurements. Moreover, these gels form simply by mixing, eliminating the need for additional cross-linking chemicals or conditions such as UV light exposure, which holds promise for their use in cell culture and therapeutic applications. We found gelation to correlate with β-sheet formation, which revealed the importance of peptide secondary structure in stereocomplex cross-linking. We also note the resemblance of rheological properties of these gels to that of spider silk, which undergoes strain stiffening as β-sheets unfold prior to eventual fracture of crystalline β-sheet peptide domains.^54,55^ Although we expect that the crystalline KYFIL stereocomplexes constrains the ability of the hydrogels to fully recover after application and removal of high strains, they still exhibit dynamic behavior to a limited extent. These findings highlight exciting new questions about the role of peptide design on the balance between stereocomplex crystallinity, which likely underpins increases in stiffness and stability, and hydrogel dynamic nature. Furthermore, the stereocomplexed hydrogels confer proteolytic stability relative to the l-conjugates, showcasing the opportunity to control hydrogel lifetime with stereocomplex cross-links. Collectively, the methodology and the results of this study establish a framework with which we can leverage the expansive design space and functionality of peptides to engineer polymer hydrogels that meet a wide range of needs in biomanufacturing, medicine, and biology.

## Author contributions

I. D. and R. A. L. conceived the idea and designed experiments. I. D. conducted all experiments and associated analysis except for DOSY and XRD. J. P. contributed acquiring HPLC chromatograms and K. R. performed and analyzed the DOSY experiments. D. R. M. and D. A. D. acquired and analyzed XRD data. I. D. and R. A. L. wrote the manuscript with input from all authors. R. A. L. supervised the work.

## Conflicts of interest

There are no conflicts to declare.

## Supplementary Material

SC-016-D5SC00251F-s001

## Data Availability

The data supporting this article have been included as part of the ESI.†

## References

[cit1] Worch J. C., Prydderch H., Jimaja S., Bexis P., Becker M. L., Dove A. P. (2019). Nat. Rev. Chem..

[cit2] Saklani R., Domb A. J. (2024). ACS Omega.

[cit3] Slager J., Domb A. J. (2003). Adv. Drug Delivery Rev..

[cit4] Tsuji H. (2005). Macromol. Biosci..

[cit5] Tsuji H. (2016). Adv. Drug Delivery Rev..

[cit6] Ren J. M., Lawrence J., Knight A. S., Abdilla A., Zerdan R. B., Levi A. E., Oschmann B., Gutekunst W. R., Lee S.-H., Li Y., McGrath A. J., Bates C. M., Qiao G. G., Hawker C. J. (2018). J. Am. Chem. Soc..

[cit7] Ren J. M., Knight A. S., van Ravensteijn B. G. P., Kohl P., Bou Zerdan R., Li Y., Lunn D. J., Abdilla A., Qiao G. G., Hawker C. J. (2019). J. Am. Chem. Soc..

[cit8] Abdilla A., Dolinski N. D., de Roos P., Ren J. M., van der Woude E., Seo S. E., Zayas M. S., Lawrence J., Read de Alaniz J., Hawker C. J. (2020). J. Am. Chem. Soc..

[cit9] Longo J. M., DiCiccio A. M., Coates G. W. (2014). J. Am. Chem. Soc..

[cit10] Auriemma F., De Rosa C., Di Caprio M. R., Di Girolamo R., Ellis W. C., Coates G. W. (2015). Angew. Chem., Int. Ed..

[cit11] Auriemma F., De Rosa C., Di Caprio M. R., Di Girolamo R., Coates G. W. (2015). Macromolecules.

[cit12] Hennink W. E., De Jong S. J., Bos G. W., Veldhuis T. F. J., van Nostrum C. F. (2004). Int. J. Pharm..

[cit13] de Jong S. J., van Eerdenbrugh B., van Nostrum C. F., Kettenes-van den Bosch J. J., Hennink W. E. (2001). J. Controlled Release.

[cit14] De Jong S. J., De Smedt S. C., Demeester J., Van Nostrum C. F., Kettenes-van Den Bosch J. J., Hennink W. E. (2001). J. Controlled Release.

[cit15] Tutoni G. G., McDonald S. M., Zhong R., Lu A., Huang T. J., Becker M. L. (2024). J. Am. Chem. Soc..

[cit16] Nagy K. J., Giano M. C., Jin A., Pochan D. J., Schneider J. P. (2011). J. Am. Chem. Soc..

[cit17] Nagy-Smith K., Beltramo P. J., Moore E., Tycko R., Furst E. M., Schneider J. P. (2017). ACS Cent. Sci..

[cit18] Swanekamp R. J., DiMaio J. T. M., Bowerman C. J., Nilsson B. L. (2012). J. Am. Chem. Soc..

[cit19] Swanekamp R. J., Welch J. J., Nilsson B. L. (2014). Chem. Commun..

[cit20] Duti I. J., Florian J.
R., Kittel A. R., Amelung C. D., Gray V. P., Lampe K. J., Letteri R. A. (2023). J. Am. Chem. Soc..

[cit21] Bomb K., Zhang Q., Ford E. M., Fromen C. A., Kloxin A. M. (2023). ACS Macro Lett..

[cit22] Tutoni G., Lu A., Bonacci M. E., Jun Huang T., Becker M. L. (2025). Biomacromolecules.

[cit23] Tutoni G., Becker M. L. (2020). Macromolecules.

[cit24] Mao H., Shan G., Bao Y., Wu Z. L., Pan P. (2016). Soft Matter.

[cit25] Hiemstra C., Zhong Z., Dijkstra P. J., Feijen J. (2005). Macromol. Symp..

[cit26] Hiemstra C., Zhong Z., Li L., Dijkstra P. J., Feijen J. (2006). Biomacromolecules.

[cit27] Michalski A., Socka M., Brzeziński M., Biela T. (2018). Macromol. Chem. Phys..

[cit28] Corrigan D. O., Healy A. M., Corrigan O. I. (2002). Int. J. Pharm..

[cit29] Van Tomme S. R., Mens A., Van Nostrum C. F., Hennink W. E. (2008). Biomacromolecules.

[cit30] Gray V. P., Amelung C. D., Duti I. J., Laudermilch E. G., Letteri R. A., Lampe K. J. (2022). Acta Biomater..

[cit31] Ding L., Jiang Y., Zhang J., Klok H.-A., Zhong Z. (2018). Biomacromolecules.

[cit32] Clarke D. E., Pashuck E. T., Bertazzo S., Weaver J. V. M., Stevens M. M. (2017). J. Am. Chem. Soc..

[cit33] Collier J. H., Messersmith P. B. (2004). Adv. Mater..

[cit34] Stahl P. J., Romano N. H., Wirtz D., Yu S. M. (2010). Biomacromolecules.

[cit35] Webber M. J., Appel E. A., Meijer E. W., Langer R. (2016). Nat. Mater..

[cit36] Raskatov J. A., Schneider J. P., Nilsson B. L. (2021). Acc. Chem. Res..

[cit37] Urban J. M., Ho J., Piester G., Fu R., Nilsson B. L. (2019). Molecules.

[cit38] Tang J. D., Mura C., Lampe K. J. (2019). J. Am. Chem. Soc..

[cit39] Tang J. D., Roloson E. B., Amelung C. D., Lampe K. J. (2019). ACS Biomater. Sci. Eng..

[cit40] Henise J., Hearn B. R., Ashley G. W., Santi D. V. (2015). Bioconjugate Chem..

[cit41] Aimetti A. A., Machen A. J., Anseth K. S. (2009). Biomaterials.

[cit42] Tan H., DeFail A. J., Rubin J. P., Chu C. R., Marra K. G. (2010). J. Biomed. Mater. Res., Part A.

[cit43] Northrop B. H., Frayne S. H., Choudhary U. (2015). Polym. Chem..

[cit44] Nair D. P., Podgórski M., Chatani S., Gong T., Xi W., Fenoli C. R., Bowman C. N. (2014). Chem. Mater..

[cit45] Caballero-Herrera A., Nordstrand K., Berndt K. D., Nilsson L. (2005). Biophys. J..

[cit46] Jahan I., Nayeem S. M. (2018). ACS Omega.

[cit47] Steinke N., Gillams R. J., Pardo L. C., Lorenz C. D., McLain S. E. (2016). Phys. Chem. Chem. Phys..

[cit48] Stumpe M. C., Grubmüller H. (2007). J. Am. Chem. Soc..

[cit49] Sagle L. B., Zhang Y., Litosh V. A., Chen X., Cho Y., Cremer P. S. (2009). J. Am. Chem. Soc..

[cit50] Elder A. N., Dangelo N. M., Kim S. C., Washburn N. R. (2011). Biomacromolecules.

[cit51] Groves P. (2017). Polym. Chem..

[cit52] Ollier R. C., Xiang Y., Yacovelli A. M., Webber M. J. (2023). Chem. Sci..

[cit53] Ollier R. C., Webber M. J. (2024). Biomacromolecules.

[cit54] Chan N. J.-A., Gu D., Tan S., Fu Q., Pattison T. G., O'Connor A. J., Qiao G. G. (2020). Nat. Commun..

[cit55] Du N., Yang Z., Liu X. Y., Li Y., Xu H. Y. (2011). Adv. Funct. Mater..

[cit56] Xu J., Jiang Y., Gao L. (2023). J. Mater. Chem. B.

[cit57] Duvaud S., Gabella C., Lisacek F., Stockinger H., Ioannidis V., Durinx C. (2021). Nucleic Acids Res..

