# Supplementary material for: Peptide stereocomplex cross-links for polymer hydrogels
Source: Chem Sci. 2025 Jun 2;16(26):11931–8. doi: 10.1039/d5sc00251f (PMC12129089; doi:10.1039/d5sc00251f)
Supplement: SC-016-D5SC00251F-s001 [file SC-016-D5SC00251F-s001.pdf]

Supplementary Information (SI) for Chemical Science.
This journal is © The Royal Society of Chemistry 2025

Supporting information

Peptide stereocomplex cross-links for polymer hydrogels

Authors: Israt Jahan Duti,[a] Jonathan Paul,[a] Keelin S. Reilly,[b] Darren R. Miller,[a] Diane A. Dickie,[c] Rachel A. Letteri[1*a]

[a]Department of Chemical Engineering, University of Virginia, Charlottesville, VA 22903

[b]Department of Biomedical Engineering, University of Virginia, Charlottesville, VA 22904

[c]Department of Chemistry, University of Virginia, Charlottesville, VA 22904

*Corresponding author

Table of Contents

1. Materials and methods 2

1.1. Materials 2

1.2. Peptide synthesis 3

1.3. Peptide purification 3

1.4. Peptide conjugation with polymer 4

1.4.1 Model conjugation with 1-arm PEG 5k-maleimide 4

1.4.2. Conjugation with 4-arm PEG 20k-maleimide 4

1.5. Characterization of peptides, polymers, and conjugates 5

1.5.1. Matrix-assisted laser desorption-ionization time-of-flight (MALDI-TOF): 5

1.5.2. ^{1}H nuclear magnetic resonance (NMR) spectroscopy 5

1.5.3. Diffusion NMR spectroscopy (DOSY) 5

1.5.4. Circular dichroism (CD) spectroscopy 6

1.5.5. Fourier transformed infrared (FTIR) spectroscopy 7

1.5.6. High pressure liquid chromatography (HPLC) 7

1.5.7. Size exclusion chromatography (SEC) 7

1.6. Hydrogel formation 8

1.7. Rheology 8

1.8. X-ray diffraction (XRD) experiments 9

1.9. Proteolytic stability measurements 9

2. Results and discussions 10

2.1. Peptide purification and characterization 10

2.1.1. Peptide purification 10

2.1.2. MALDI-TOF mass spectrometry 12

2.1.3. ^{1}H NMR spectroscopy 12

2.1.4. Circular Dichroism spectroscopy 14

2.1.5. Fourier transformed infrared spectroscopy 15

2.2. Conjugation of peptide and polymer 15

2.2.1. Model peptide-polymer conjugation 15
2.2.2. Peptide conjugation with 4-arm PEG-maleimide 17
2.2.3. HPLC chromatograms of conjugates 18
2.2.4. Size exclusion chromatography 20
2.2.5. ^{1}H NMR spectroscopy 21
2.2.6. Diffusion NMR spectroscopy 24
2.2.7. CD spectroscopy 27
2.3. Hydrogel formation 28
2.3.1. Images of conjugates in PBS 28
2.3.2. IR spectroscopy 29
2.4. Rheology 30
2.4.1. Amplitude sweeps at 5% (w/v) 30
2.4.2. Amplitude sweeps at 7.5% (w/v) 31
2.4.3. Amplitude sweeps at 10% (w/v) 32
2.4.4. Frequency sweeps at 5% (w/v) 33
2.4.5. Frequency sweeps at 7.5% (w/v) 34
2.4.6. Frequency sweeps at 10% (w/v) 35
2.4.7. Stress recovery of hydrogels at 7.5% (w/v) 36
2.4.8. Stress recovery of hydrogels at 10% (w/v) 37
2.4.9. Stress recovery experiments on hydrogels over longer times 38
2.5. X-ray diffraction patterns 39
2.6. Proteolytic stability measurement 41
2.6.1. HPLC chromatograms of controls 41
2.6.2. HPLC chromatograms of L-conjugates 42
2.6.3. HPLC chromatograms of D-conjugates 43
2.6.4. HPLC chromatograms of 1:1 L:D-conjugates 44
3. References 45

1. Materials and methods

1.1. Materials

Rink amide resin (0.5 mmol/g loading, 100–200 mesh), Oxyma Pure, and 9-fluorenylmethyloxycarbonyl (Fmoc)-protected amino acids (Fmoc-L-Lys-OH and Fmoc-D-Lys-OH, Fmoc-L-Tyr(tBu)-OH and Fmoc-D-Tyr(tBu)-OH, Fmoc-L-Phe-OH and Fmoc-D-Phe-OH, Fmoc-L-Ile-OH and Fmoc-D-Ile-OH, Fmoc-L-Leu-OH and Fmoc-D-Leu-OH, Fmoc-L-Cys(Trt)-OH and Fmoc-D-Cys(Trt)-OH) were purchased from Advanced ChemTech. *N*,*N*-dimethylformamide (DMF, ACS reagent, ≥99.8%), dichloromethane (DCM, ≥99.5%), *N*,*N′*-diisopropyl carbodiimide (DIC, ≥99%), piperidine (≥99%), trifluoroacetic acid (TFA, ≥99%), triisopropylsilane (TIPS, ≥98%), 2,2′-(ethylenedioxy) diethanethiol (DODT, ≥95%), deuterium oxide (99.9%), diethyl ether (98%, containing ~2% ethanol and ~10 ppm butylated hydroxytoluene as inhibitors), dithiothreitol (DTT,

≥98%), phosphate-buffered saline tablets, acetonitrile (HPLC grade, ≥99.9%), 2,2,2-trifluoroethanol (TFE, ≥99%), sodium trifluoroacetate (NaT-FAc, ≥98%), maleimide-functionalized 1-arm polyethylene glycol maleimide (1-arm PEG-mal, average M_n = 5,000 g/mol) and 4-arm polyethylene glycol maleimide (4-arm PEG-mal, average M_n = 20,000 g/mol), and Proteinase K (from Tritirachium album) were obtained from Sigma-Aldrich. Spectra/Por® 7 dialysis membranes (molecular weight cut-off = 3.5 kDa, diameter = 28.6 mm, flat length = 45 mm, in 0.1% sodium azide as a preserving agent) were purchased from VWR (USA). Deionized water (18.2 MΩ cm) was acquired from a Thermo Scientific Smart2Pure water purification system. All chemicals were used without further purification.

1.2. Peptide synthesis

We synthesized peptides by solid phase peptide synthesis (SPPS) method on a Rink amide resin at 0.25 mmol scale with a CEM Liberty Blue™ microwave-assisted peptide synthesizer. Resin was swollen with dimethylformamide and the Fmoc groups on the resin were removed with 20/80 (v/v)% piperidine/DMF. Coupling of the C-terminal amino acid to the solid support involved addition of Fmoc-protected amino acid (0.2 M in DMF) and the coupling agents diisopropylcarbodiimide (1 M in DMF) and Oxyma Pure (1 M in DMF) to the reaction vessel and heating at 90 °C for 4 min. Fmoc deprotection and coupling cycles were repeated to construct the desired peptide sequence from the C-terminus to the N-terminus on the resin. After synthesis, the peptide-loaded resin was treated with 20 mL 92.5/2.5/2.5/2.5 (v/v/v/v)% trifluoroacetic acid/ triisopropylsilane/ 2,2′-(ethylenedioxy) diethanethiol/ DI water for 3 h to simultaneously cleave the peptide from the resin and remove protecting groups from amino acid side chains. The peptides were then precipitated into cold (-20 °C) diethyl ether (120 mL) and isolated by centrifugation. The precipitated peptides were washed again with fresh cold (-20°C) diethyl ether (120 mL) and dried under vacuum, and then stored at −20 °C as powders until ready for purification. The percent recovery of the peptides was 80-85%.

1.3. Peptide purification

For purification, the peptides (~40 mg) were dissolved in 10 mL 20/80 (v/v)% acetonitrile/water solution with 0.1 (v/v)% TFA and filtered by 13 mm syringe filter with 0.45 μm polytetrafluoroethylene (PTFE) membrane. The peptides were then injected into a Waters preparative HPLC system equipped with a photodiode array detector and a reversed-phase column (30 mm x 150 mm, 5 μm). A linear gradient from 20/80 (v/v)% acetonitrile/water with 0.1 (v/v)% TFA to 95/5 (v/v)% acetonitrile/water with 0.1 (v/v)% TFA was applied over 18 min at 35°C

with a flowrate of 25.52 mL/min. Elution of peptide was monitored by absorbance at 214 nm. The peptide eluted between 11 to 13 min and the collected fractions were lyophilized to give a fluffy white solid that we stored at −20 °C until further use. The percentage recoveries of peptides from the column ranged from 50-65%.

The purified peptides were characterized by mass spectrometry, ^{1}H nuclear magnetic resonance (NMR) spectroscopy, high pressure liquid chromatography (HPLC), size exclusion chromatography (SEC), circular dichroism (CD) spectroscopy and Fourier transformed infrared (FTIR) spectroscopy, as detailed in the section 1.5.

Probing disulfide bond formation with DTT

After purifying and storing in -20°C freezer, HPLC chromatograms of KYFILC peptides showed a new peak adjacent to the peptide peak. Suspecting dimer formation among the peptides via disulfibe bond at their thiol group, we used the reducing agent dithiothreitol (DTT) to break the bonds. We dissolved DTT (19.8 mg) in 150 mM PBS (6.5 mL) and L-KYFILC (4.4 mg) in 150 mM PBS (2.2 mL), then mixed the DTT solution (1.1 mL) with the L-KYFILC solution (2.2 mL) on a stir plate for 1 h, prior to injecting samples onto the HPLC column. The disappearance of the smaller adjacent peak upon exposure to DTT (Figure S3) allowed us to attribute this peak to disulfide-linked peptide.

1.4. Peptide conjugation with polymer

1.4.1 Model conjugation with 1-arm PEG 5k-maleimide

In the model conjuagtion reaction, L-KYFILC peptides were attached to 1-arm PEG5k-maleimide using a thiol-maleimide click reaction. Peptides and polymer were dissolved individually in 150 mM PBS to prepare 1 mM solutions, which were then mixed in a 1:1 volumetric ratio and stirred 6 h at room temperature. We used HPLC to monitor the conjugation reaction. We note that we found no reaction between KYFILC and polymers in 150 mM PBS. To disrupt the hydrogen bonds of the peptides and facilitate the reaction, we next performed the reaction using the same conditions in presence of 7 M urea. HPLC showed the consumption of peptide, indicative of successful conjugation (Figure S9).

1.4.2. Conjugation with 4-arm PEG 20k-maleimide

Both L- and D-KYFILC peptides were conjugated to 4-arm PEG 20k-maleimide using a thiol-maleimide click reaction. Peptides and polymer were dissolved individually in 7M urea in 150 mM PBS (peptides: 66 mg in 65 mL and polymer: 260 mg in 13 mL) to prepare 1 mM solutions,

which were mixed in a 5:1 volumetric ratio and stirred 6 h at room temperature. The reaction mixture was dialyzed against deionized water with a 3.5 kDa MWCO dialysis membrane to remove excess peptide, urea, and salt. Dialysis was performed for 24 h, changing the water 3 times. The conjugates were then frozen in liquid nitrogen, lyophilized, and stored at −20 °C until ready for use. We used HPLC, ^{1}H NMR spectroscopy, diffusion NMR spectroscopy, SEC, CD and FTIR spectroscopy to confirm conjugation and characterize the conjugates, as described in section 1.5.

1.5. Characterization of peptides, polymers, and conjugates

1.5.1. Matrix-assisted laser desorption-ionization time-of-flight (MALDI-TOF):

We acquired matrix-assisted laser desorption-ionization time-of-flight mass spectrometry to determine the molar masses of synthesized peptides and conjugates. The samples (5 mg) were dissolved in 20/80 (v/v)% acetonitrile/water solvent (2.5 mL) and the sample solution (1 μL) was coated with a α-cyano-4-hydroxycinnamic acid (CHCA) matrix (1 μL) on a plate and was air dried before measurement. Mass spectra were acquired with Shimadzu MALDI-8030 system with a 200 Hz solid-state laser (355 nm). The instrument was calibrated with a standard MALDI calibration kit (TOFMix, Shimadzu) covering a 300 Da to 4000 Da molecular weight range.

1.5.2. ^{1}H nuclear magnetic resonance (NMR) spectroscopy

We obtained ^{1}H NMR spectra of peptides, polymers, and conjugates to characterize their primary structure and confirm the conjugation of peptide and polymer. The spectra were acquired at room temperature on a Bruker Avance III 800 spectrometer in deuterium Oxide (D_2O). NMR spectra were acquired at 1% (w/v) (10 mg/mL) with 16 scans for peptides and at 1.5% (w/v) (20 mg/1 mL) with 32 scans for polymers and conjugates. All spectra were analyzed in MestReNova software to verify the structure of the peptides and conjugates from the chemical shifts and the integration of protons.

1.5.3. Diffusion NMR spectroscopy (DOSY)

We ran diffusion ordered NMR spectroscopy experiments of peptides, polymers, and conjugates at 1% (w/v) in D_2O on a Bruker Neo 600 spectrometer using a standard Bruker 'stebpgp1s' pulse sequence to gauge their size in solution by measuring the diffusion coefficients.[1] We first collected a standard 1D spectra of the sample to set the spectral width (SW) and offset frequency (O1), which is the center of the spectra. The 1D spectra was collected using 4 scans and a relaxation delay of 5 s. We then collected a spectrum at low gradient strength of 2% (i.e.,

the gradient strength that attenuates just 2% of a selected peak), followed by a spectrum at high gradient strength of 95% to determine the optimal delta parameters. We used the set spectral width (8.50 ppm), offset frequency (3.26 ppm), and number of points in the time domain of the FID (TD) = 8192 points. Our goal was to set a diffusion time, Δ, and gradient pulse duration, δ, that makes the slowest diffusing peak in our sample attenuate about 95% between the low and high gradient strengths.

The 4-arm PEG-mal was used to determine the optimal delta parameters and ensure the curve would fit well for the largest sample, resulting in a gradient pulse duration δ = 3900 μs and delay time between gradient pulses (or diffusion time) Δ = 0.35 s. The δ was set through the P30 parameter = δ *0.5 and was set to 1950 μs. Once we set our parameters, we ran the DOSY experiment with a minimum gradient strength of 2%, a maximum gradient strength of 95%, and a total of 16 intermediate strengths along a linear ramp type for each sample, keeping the identified parameters constant for each of the samples. Using the DOSY/ROSY transform function and the Peak Fit method in MestReNova (Mestre Lab Research, S.L.U.), we determined the diffusion coefficients of the various peaks in the peptides, polymers, and conjugates (shown in Figure S18), and calculated the average and standard deviation of the peak diffusion coefficients that are shown in the bar charts (Figure S19).

1.5.4. Circular dichroism (CD) spectroscopy

To confirm peptide stereochemistry, we recorded circular dichroism (CD) spectra of L-, 1:1 L:D-, and D-KYFILC solutions 0.1% (w/v) in ultrapure water on a Jasco J-1500 CD spectrophotometer. We chose these conditions, rather than those used for gelation, because spectra taken in PBS or at concentrations higher than 0.1% (w/v) in water led to low signal to noise ratios. Solutions of L- and D-peptides were prepared separately by dissolving peptides (5 mg) in ultrapure water (5 mL) at 0.1% (w/v). For stereocomplexed KYFILC, peptide stock solutions were mixed (by stirring) at 1:1 (1 mL:1 mL) volumetric ratio of L- to D-peptides. For the conjugates, the samples were prepared at 0.59% (w/v) to keep the equivalent peptide concentration 0.1% (w/v) in the conjugates. CD spectra were acquired in a 0.1 mm path length quartz cuvette from 250 nm to 190 nm at a continuous scan speed of 50 nm/min at 25°C. Three scans were obtained for each solution, and each scan was corrected with a background ultrapure water scan. CD data were reported as ellipticity (θ in millidegrees) vs wavelength (nm).

1.5.5. Fourier transformed infrared (FTIR) spectroscopy

To determine secondary structure of the peptides and conjugates, we acquired their Fourier transform infrared (FTIR) spectra. L- and D-KYFILC peptides were dissolved in ultrapure water to prepare stock solutions at 3.3% (w/v). For samples composed of purely L-KYFILC or purely D-KYFILC, 10X PBS was added to peptide stock solution to bring the final peptide concentration to 3% (w/v) in 1X PBS. For the 1:1 L:D blend, peptide stock solutions were mixed by stirring at a 1:1 L: D volumetric ratio. The mixture was then similarly diluted with 10X PBS. The final concentrations of all the peptide samples were 3% (w/v) in 1X PBS. For the conjugates, sample of L-, 1:1 L:D-, and D-conjugates were prepared similarly at 3, 5, 7.5, 10 and 15% (w/v) in 1X PBS. After allowing the samples to sit 30 min following preparation, IR spectra were acquired on a PerkinElmer 400 FT-IR spectrometer equipped with an attenuated total reflectance (ATR) accessory. We collected 4 scans per sample at 1 cm^{-1} resolution, and subtracted the background spectra (1X PBS). Data were reported as % transmittance (T) and later converted to absorbance and plotted against wavenumber (cm^{-1}).

1.5.6. High pressure liquid chromatography (HPLC)

High pressure liquid chromatography was performed to determine the purity of the peptides and to monitor the conjugation reactions. We dissolved the samples (5 mg peptide, polymer, or conjugate) in ultrapure water in 0.1% TFA (2.5 mL) at 0.2% (w/v) and then filtered these solutions thorough 13 mm syringe filters with 0.45 μm PTFE membranes. Chromatograms were acquired on a Waters Alliance e2695 XC HPLC System equipped with a photodiode array detector and an XBridge C18 reverse-phase column (4.6 x 50 mm, 3.5 mm particle size). The samples were eluted with a linear elution gradient from 0/100 (v/v)% acetonitrile/water with 0.1 (v/v)% TFA to 95/5 (v/v)% acetonitrile/water with 0.1 (v/v)% TFA over 10 min at 35°C with a flowrate of 1 mL/min. The elution was monitored by the absorbance at 214 nm wavelength and the purity was calculated from the relative area of conjugate peaks in HPLC traces, excluding solvent peaks. The conjugations were monitored by comparing the elution times of the peptides and polymer to those in the conjugation mixtures.

1.5.7. Size exclusion chromatography (SEC)

To further characterize the conjugation reactions, SEC was conducted with EcoSEC GPC System from Tosoh Bioscience equipped with a refractive index detector and two TSKgel

SuperAWM-H mixed-bed columns (6 mm × 15 cm, 9 µm diameter beads) in series with a TSKgel SuperAWM-H mixed-bed guard column (4.6 mm × 3.5 cm, 9 µm diameter beads). Samples (5 mg peptide, polymer and conjugate) were dissolved at 0.2% (w/v) in TFE with 0.02 M sodium trifluoroacetate (2.5 mL) and then filtered thorough 13 mm syringe filter with 0.45 µm PTFE membrane. Trifluoroethanol with 0.02 M sodium trifluoroacetate was used as the eluent. The elution times were compared to confirm the conjugation.

1.6. Hydrogel formation

Gelation experiments were conducted at 5, 7.5, 10% (w/v) in 1X PBS to determine the critical gelation concentrations of the L-, 1:1 L:D, and D- conjugates. For each concentration, L- and D-conjugates were first dissolved in ultrapure water to prepare stock solutions at a higher concentration than required. For hydrogels composed of purely L- conjugates or purely D-conjugates, 10X PBS was added to the conjugate stock solutions to yield the final required peptide concentrations in 1X PBS without further pH correction. For 1:1 L:D blends, conjugate stock solutions were mixed by stirring at a 1:1 L: D volumetric ratio. The mixture was then similarly charged with 10X PBS. After 24 h of preparation, gel formation and critical gelation concentration was assessed by vial inversion test and rheology.

1.7. Rheology

Oscillatory shear rheology was performed on samples prepared at 5, 7.5 and 10% (w/v) 24 h after preparation. Samples (50 µL) were loaded into a 25°C convection temperature device (8 mm parallel plate) on an TA instrument rheometer for measurements. We used pipettes and spatulas to load liquid and gel samples, respectively. Excess sample was scraped away from the plate with a spatula. Amplitude sweeps (1-800% strain) were conducted at a constant angular frequency of 1 rad/s and a gap height of 500 µm. Frequency sweeps (1-10 rad/s) were performed at a constant strain of 5% within the linear viscoelastic range using a gap height of 500 µm. Storage (G') and loss (G'') moduli are reported as the average modulus measured at 10 rad/s from 3 independently prepared samples, with error bars representing standard deviation. For stress recovery experiments, we used step strain measurements by applying 500% strain for 200 s followed by 5% strain for 200 s to the hydrogels at 1 rad/s frequency with this step strain sequence repeated three times.

1.8. X-ray diffraction (XRD) experiments

Powder x-ray diffraction patterns were recorded for 4-arm PEG20k-maleimide, L-conjugates, D-conjugates, and their 1:1 blends. We dissolved the samples first in ultrapure water at 11.1% (w/v) then added 10X PBS to bring the final concentration to 10% (w/v). Two sets of samples were prepared independently to determine if gelation time affected crystallinity. We waited 30 min for one set and 24 h for the other set before freezing them with liquid nitrogen, followed by lyophilization. To identify pattern features stemming from crystalline salts in PBS, we lyophilized 1X PBS to run as a control. The samples were mounted on a MiTeGen MicroLoop holder and then diffraction patterns were acquired using Bruker D8 Venture Photon III Kappa four-circle diffractometer system, equipped with an Incoatec IµS 3.0 micro-focus sealed X-ray tube (Cu Kα, λ = 1.54178 Å) and a HELIOS MX double bounce multilayer mirror monochromator. The patterns were analyzed with APEX6 Software.

1.9. Proteolytic stability measurements

To assess the impact of stereocomplexation on proteolytic stability, we incubated the L-, 1:1 L:D-, and D-conjugates in Proteinase K and monitored the amount of intact gel as a function of incubation time by HPLC. Stock solutions were prepared by solubilizing of L- and D-conjugates (60.2 mg) in ultrapure water (0.72 mL, 8.3% (w/v)). For L- and D-conjugates, 10X PBS (5.6 μL, containing 2 mg/mL Proteinase K) was added to stock solution (50 μL) to form 7.5% (w/v) hydrogels with 0.2 mg/mL Proteinase K in 1X PBS. For stereocomplexed hydrogels, L- and D-conjugate stock solutions were mixed at 1:1 volumetric ratio (25 μL L- and 25 μL D-solutions), followed by the addition of 10X PBS with Proteinase K as described for the L- and D-conjugates. Three samples were prepared for each formulation and each timepoint (1 h, 12 h) for a total of 6 samples. We also incubated 4-arm PEG maleimide polymer with enzyme 7.5% (w/v) and conjugate samples without enzyme at 7.5% (w/v) following the same procedure for control stability measurements for each timepoint. Following incubation of the hydrogels for the desired time, DMSO (2.02 mL) was added to the conjugates to dissolve them, yielding a final concentration of 2 mg/mL. The samples were filtered and injected to the HPLC instrument (details in section 1.5.6.). A linear gradient from 0/100 (v/v)% acetonitrile/water with 0.1% TFA to 70/30 (v/v)% acetonitrile/water with 0.1% TFA over 10 min was applied at 1 mL/min flowrate at 35°C, and elution of peptide and degradation products were monitored at 214 nm. Proteolytic stability was quantified by the relative integration of peptide peaks in HPLC traces, excluding solvent peaks.

2. Results and discussions

2.1. Peptide purification and characterization

2.1.1. Peptide purification

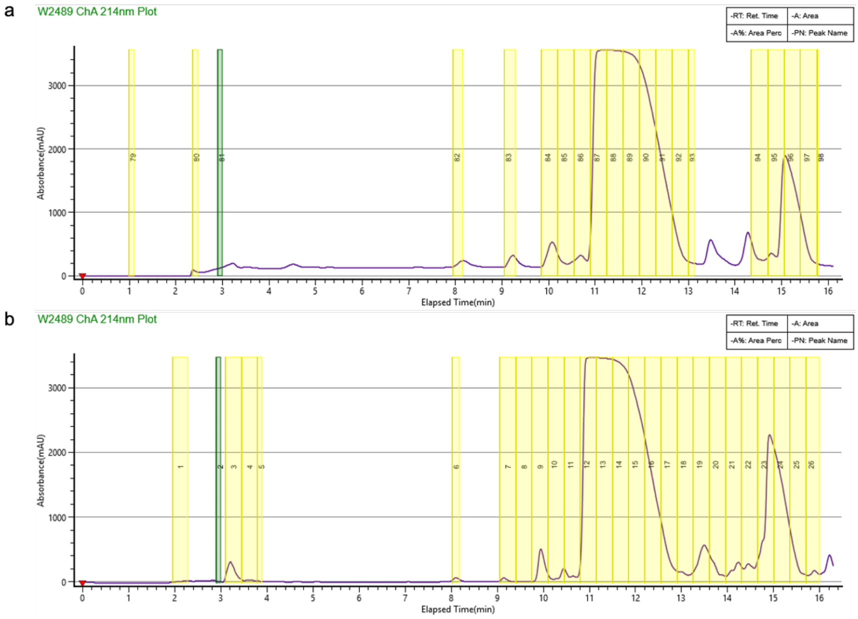

Figure S1: Preparative HPLC chromatograms of (a) L-KYFILC and (b) D-KYFILC peptides. The peptides elute between 11-13 min.

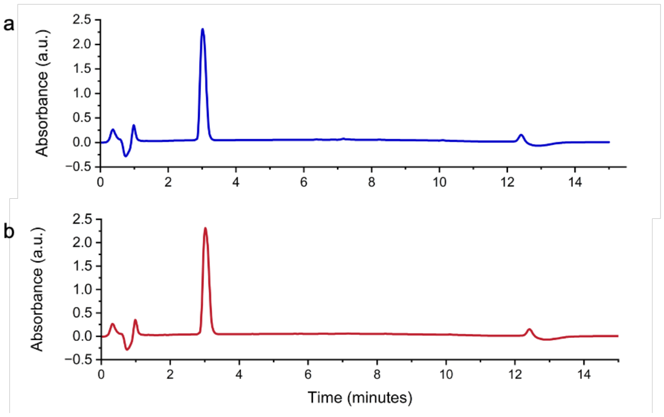

Figure S2: Analytical HPLC chromatograms of (a) L-KYFILC and (b) D-KYFILC peptides. The peptides elute at 3 min and the purities of the peptides were >95%.

After storing the purified peptides in -20°C freezer for about a month, we observed a new peak adjacent to the peptide in the HPLC chromatograms (Figure S3), As the peptides were stored in the freezer, there was less possibility of contamination. Suspecting that the thiol-containing peptides formed disulfide bonds and this dimer showed up in the HPLC traces, we stirred the reducing agent DTT with the peptides to break the disulfide bonds and acquired HPLC

trace of the mixer after 1 h. The new peak disappeared (highlighted in Figure S3), confirming the dimer formation and assuring the peptide purity as > 95%.

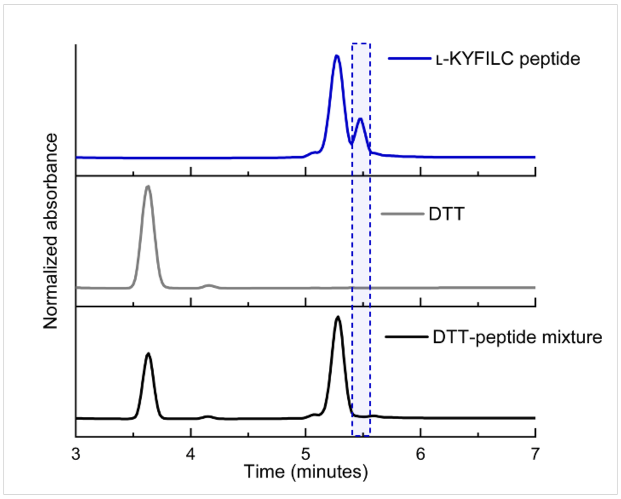

Figure S3. HPLC traces L-KYFILC peptide DTT and their mixture. The disappearance of the peak (highlighted) in the DTT-peptide mixture suggest the disulfide bond formation among the peptides.

2.1.2. MALDI-TOF mass spectrometry

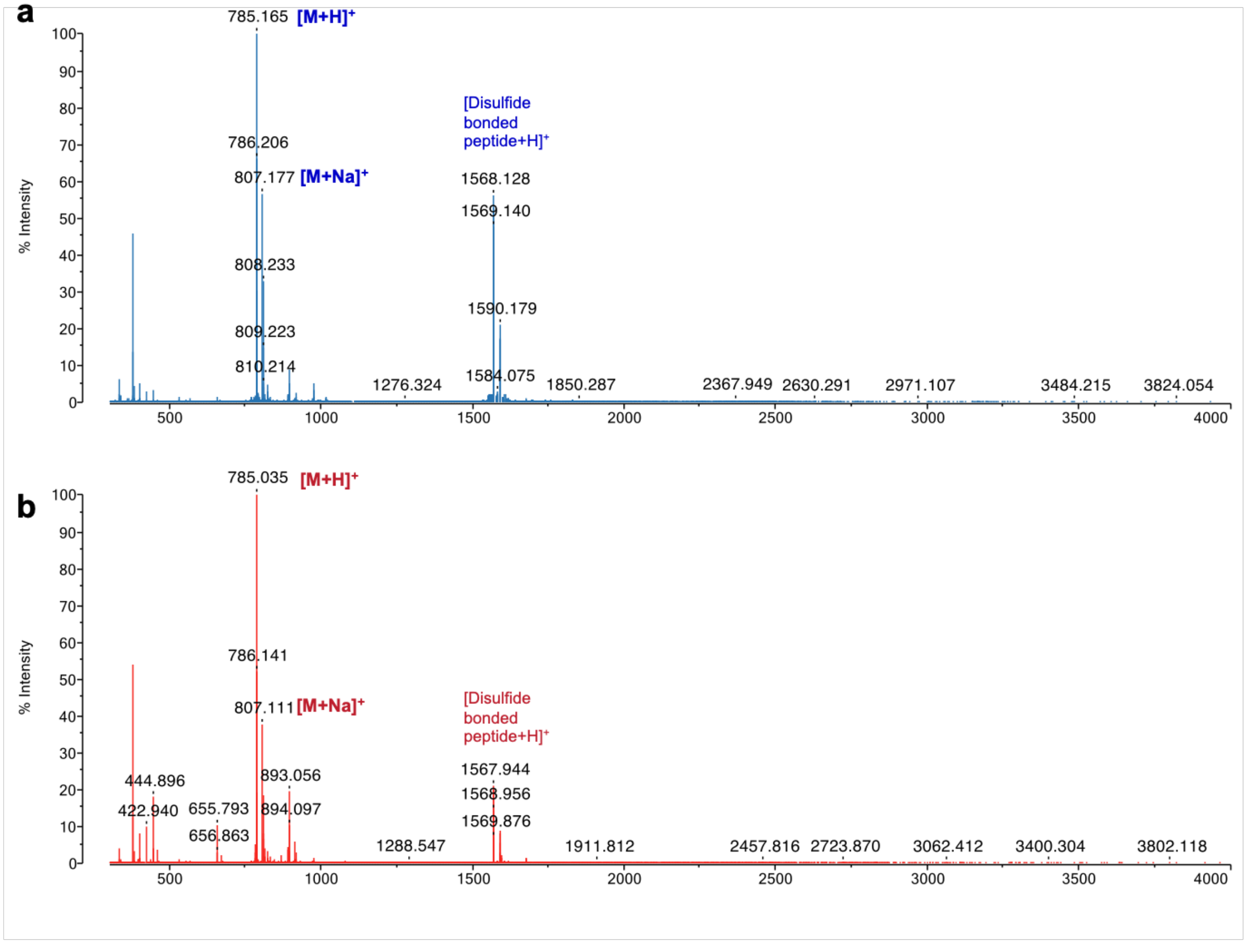

Figure S4. MALDI-TOF spectra of (a) L- KYFILC and (b) D-KYFILC

2.1.3. ^{1}H NMR spectroscopy

We used ^{1}H NMR spectroscopy to confirm the structures of the synthesized peptides. The spectra of L- and D-KYFILC are shown in Figure S5 and S6 respectively. As the amide protons exchange with D_2O, the corresponding protons do not appear in the spectra. Together, the comparison of the obtained chemical shifts in the spectra with the expected chemical shifts and relative integrations confirmed the primary structure of the KYFILC peptides.

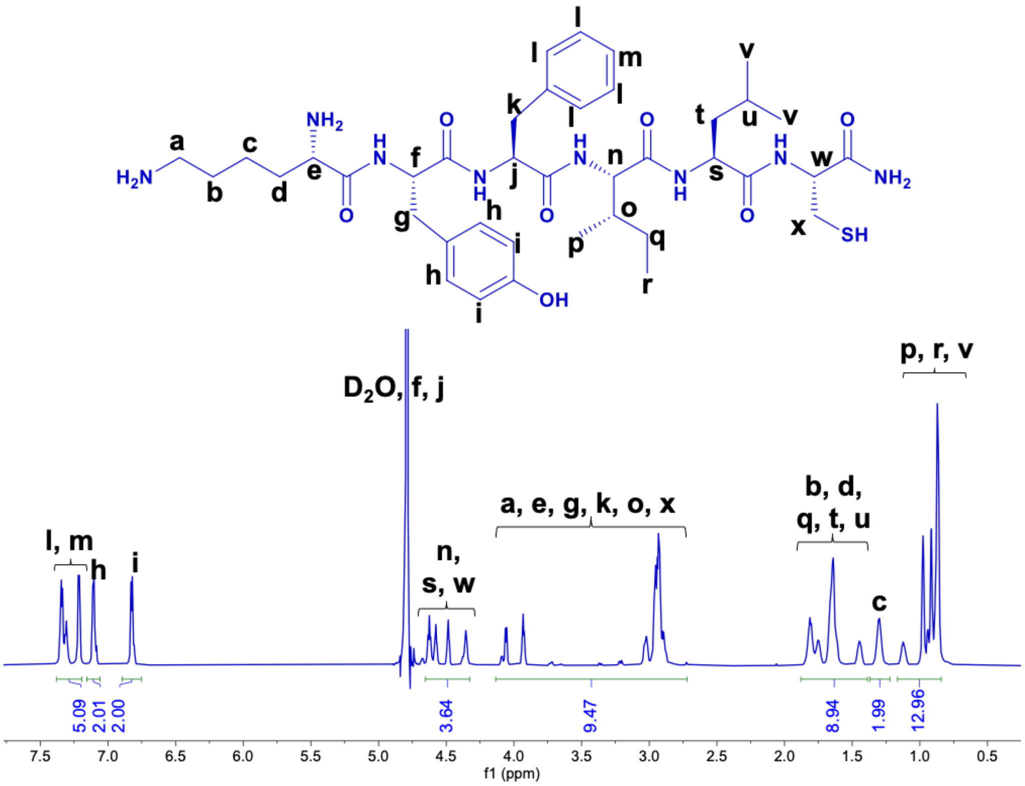

Figure S5: ^{1}H NMR spectrum (800 MHz, D_2O) of L-KYFILC peptides.

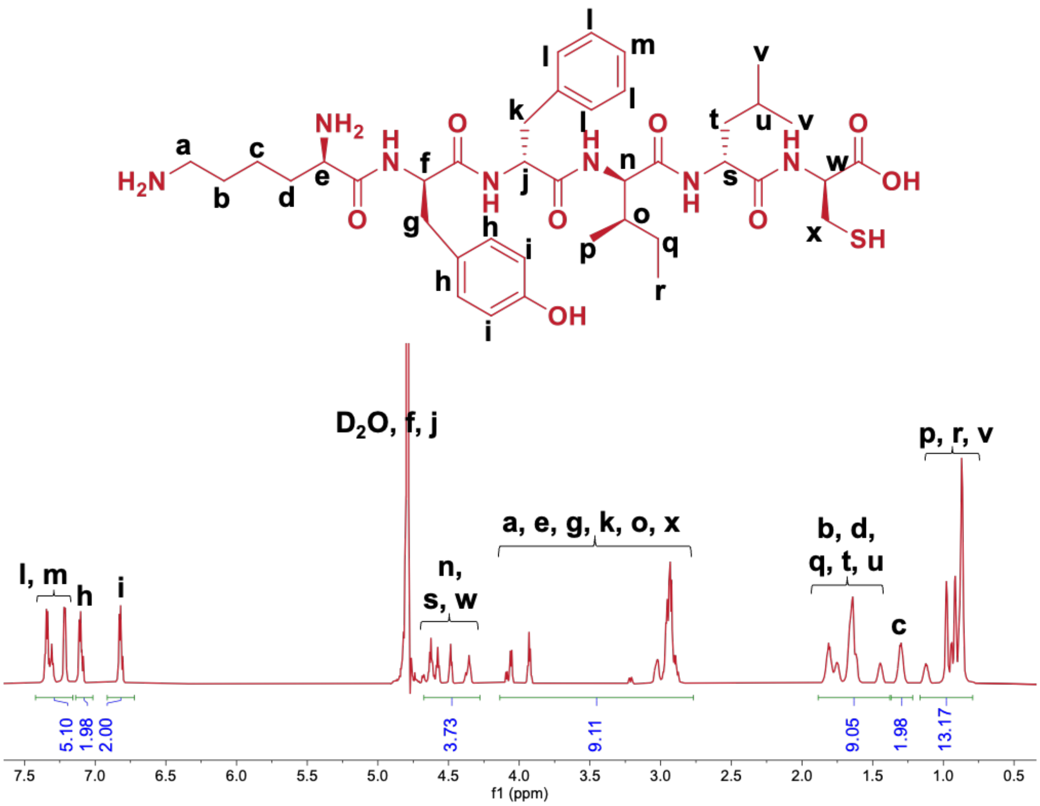

Figure S6: ^{1}H NMR spectrum (800 MHz, D_2O) of D-KYFILC peptides.

2.1.4. Circular Dichroism spectroscopy

To characterize the L- and D-configuration of the peptides and their blends, we acquired CD spectra of L-, 1:1 L:D- and D-KYFIL at 0.1% (w/v) in water. The L-KYFILC solutions produced negative ellipticity signal between 190-200 nm characteristic of L-type peptides, while D-KYFILC solutions produced the mirror image spectrum (positive ellipticity signal between 190-200 nm) characteristic of D-type peptides (Figure S7). The signals for the 1:1 blends are reduced, consistent with the negative signal from L-peptide subtracting from the positive signal of the D-peptide. Like our previous reports on KYFIL peptides[2], CD spectra do not provide evidence of β-sheets in KYFILC peptide solutions at low concentration (0.1% (w/v)). We note that CD spectra taken at concentrations higher than 0.1% (w/v) water led to low signal to noise ratios.

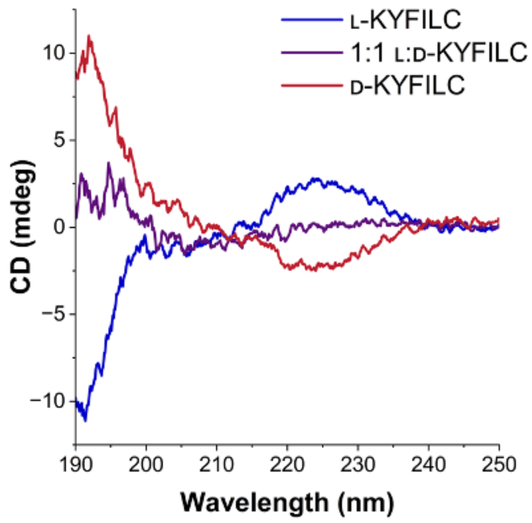

Figure S7. Circular dichroism spectra of L-, 1:1 L:D-, and D--KYFILC peptides at 0.1% (w/v) in water.

2.1.5. Fourier transformed infrared spectroscopy

To determine secondary structure of the peptides, we acquired IR spectra of unconjugated L-, 1:1 L:D-, and D-KYFILC peptide hydrogels in PBS at 3% (w/v) at pH 7.4. The amide I stretches at 1627-1636 cm^{-1} are characteristic of β-sheets and together with the broad absorbance at ~1665 cm^{-1} indicate the presence of antiparallel β-sheets in all formulations. Therefore, cysteine functionalization does not appear to affect the β-sheet secondary structure of the KYFIL peptides.

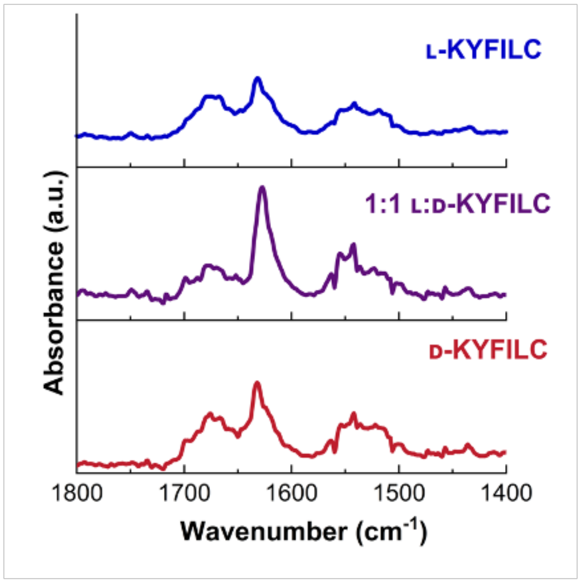

Figure S8: FTIR spectra of KYFIL hydrogel formulations (3% w/v in PBS at pH 7.4, method 1), with the peaks at 1627-1636 cm^{-1} and ~ 1665 cm^{-1} indicating the presence of antiparallel β-sheets in all hydrogels.

2.2. Conjugation of peptide and polymer

2.2.1. Model peptide-polymer conjugation

We initially performed a model conjugation in 1X PBS with 1-arm PEG5k-Maleimide and crude L-KYFILC peptide. The chromatogram (Figure S9a) of reaction mixture show both peptide (5.3 min) and polymer (6.25 min) peaks, indicating that no reaction occurred. Urea can denature peptide and protein secondary and tertiary structures at high concentrations (e.g 4 M, 8 M etc.) by disrupting hydrogen bonds. Based on the prior reports of urea facilitating the conjugation of β-sheet peptide to polymers[3–8], we conducted the conjugation in presence of 7M urea in 1X PBS. The consumption of the peptides in the reaction mixture chromatograms (Figure S9b) indicates succesful conjugation.

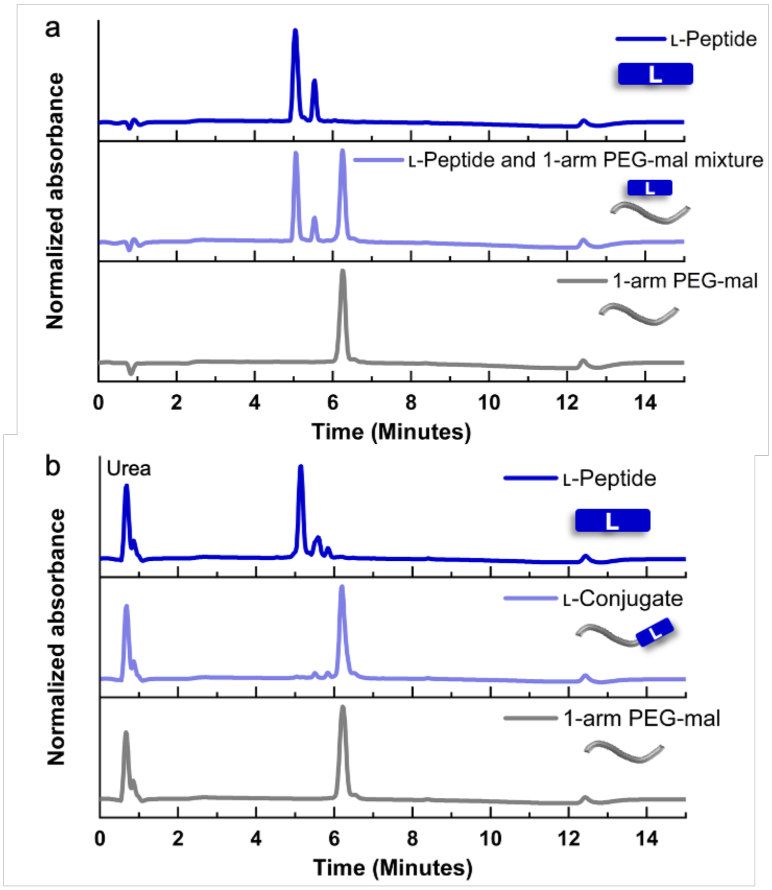

Figure S9. HPLC traces L-KYFILC peptide, 1-arm PEG 5k-maleimide and their mixtures. (a) No conjugation observed in PBS as both the peptide and polymer peaks appeared in the peptide-polymer mixture chromatogram. (b) In presence of 7M urea in PBS, disappearance of the peptide peak suggest the success of conjuagtion.

2.2.2. Peptide conjugation with 4-arm PEG-maleimide

KYFILC peptides assemble in presence of salt and thus precipitate or are partially soluble in 7M urea PBS solution. For both L- and D-KYFILC peptides, mixture of turbid peptide and clear polymer solution remain cloudy (t = 0 h in Figure S10). As the conjugation reaction progresses, the mixture becomes clear within 24 h (t = 24 h in Figure S10), suggesting successful conjugation.

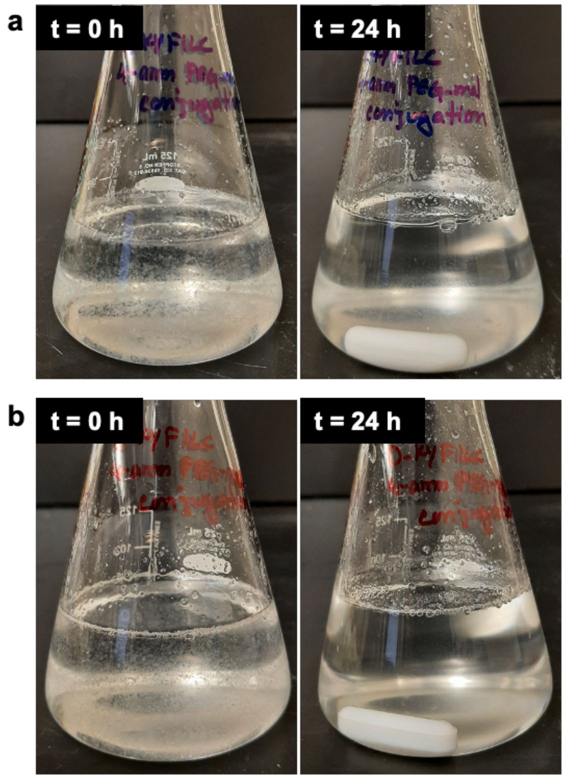

Figure S10: Mixtures of (a) L-KYFILC and (b) D-KYFILC peptides and 4-arm PEG20k-maleimide in 7M urea in 150 mM PBS at 0 h and 24 h. Immediately after mixing (t = 0 h) we observed precipitates attributed to peptide assembly and aggregation. After 24 h, the solution reclarified, indicative of peptide conjugation to the polymers.

2.2.3. HPLC chromatograms of conjugates

The conjugation reactions were also monitored by HPLC: the KYFILC peptides and 4-arm PEG 20k-maleimide elute at 5.3 min and 6.75 min respectively, indicating the polymer to be less polar relative to the peptides. When peptides conjugate to the polymer, the resulting conjugates are expected to have polarity in between the peptide and polymers and thus an intermediate elution time. In the conjugate traces, the peptide and polymer peaks (Figure S11 for L-KYFILC conjugation, Figure S12 for D-KYFILC conjuagtion) disappear and a new peak appears at 6.45 min. and thereby suggest the attachment of the peptides to polymer.

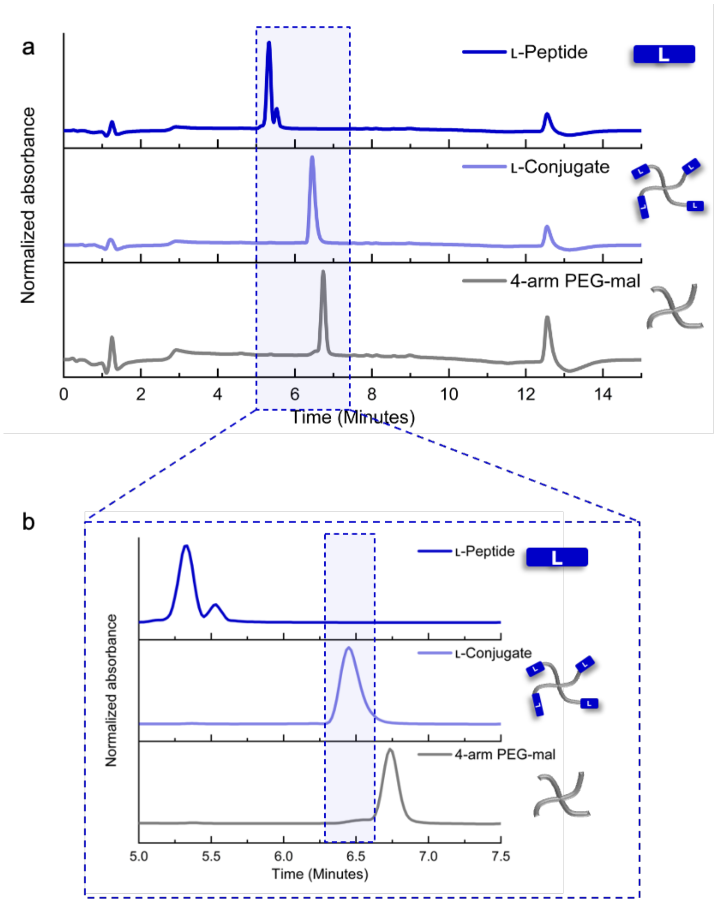

Figure S11. HPLC traces (a) full chromatograms and (b) zoomed in chromatograms of L-KYFILC peptide, 4-arm PEG 20k-maleimide and their conjugates. The disappearance of L-KYFILC and 4-arm PEG at 5.3 min and 6.75 min respectively in the conjugate traces, and the appearrance of new peak at 6.45 min (highlighted in b) suggest sucessful conjuagtion.

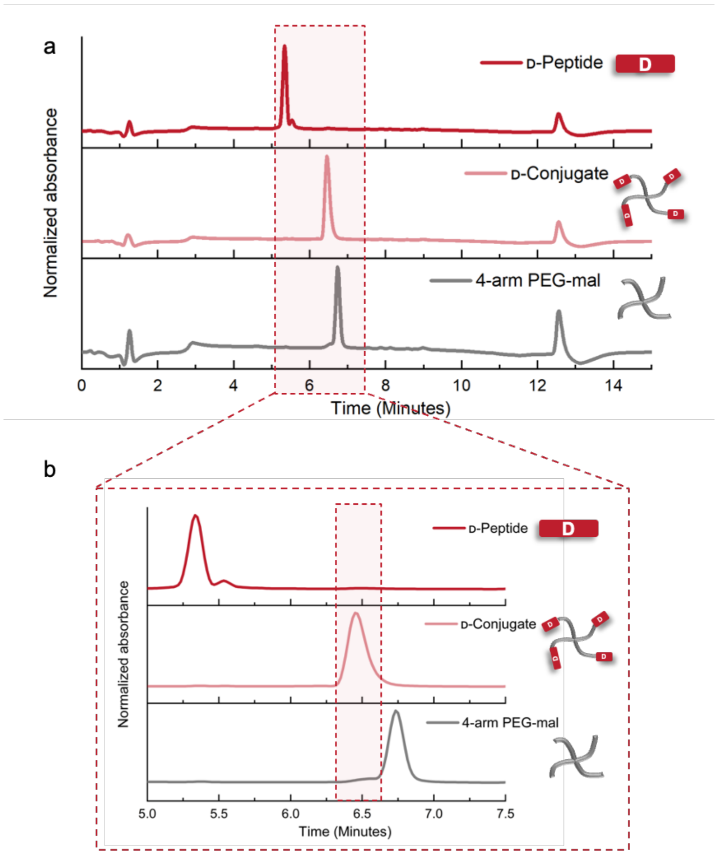

Figure S12. HPLC traces (a) full chromatograms and (b) expanded chromatograms of D-KYFILC peptide, 4-arm PEG 20k-maleimide and their conjugates. The disappearance of D-KYFILC and 4-arm PEG at 5.3 min and 6.75 min respectively in the conjugate traces, and the appearrance of new peak at 6.45 min (highlighted in b) suggest sucessful conjuagtion.

2.2.4. Size exclusion chromatography

Apart from HPLC, the conjugation reactions were also confirmed by SEC. Larger 4-arm PEG-mal eluted at 14.9 min, earlier as expected than the smaller peptides which elute at 19.1 min (Figure S13a-b). In the conjugate SEC traces, the peptide and polymer peaks disappeared, and a peak appeared with slightly smaller elution time at 14.8 min, suggesting the presence of a slightly larger conjugate. We note that the shoulder in the conjugate traces from 13.5-14.3 min may be attributed to aggregation of the conjugates in the trifluoroethanol eluent.

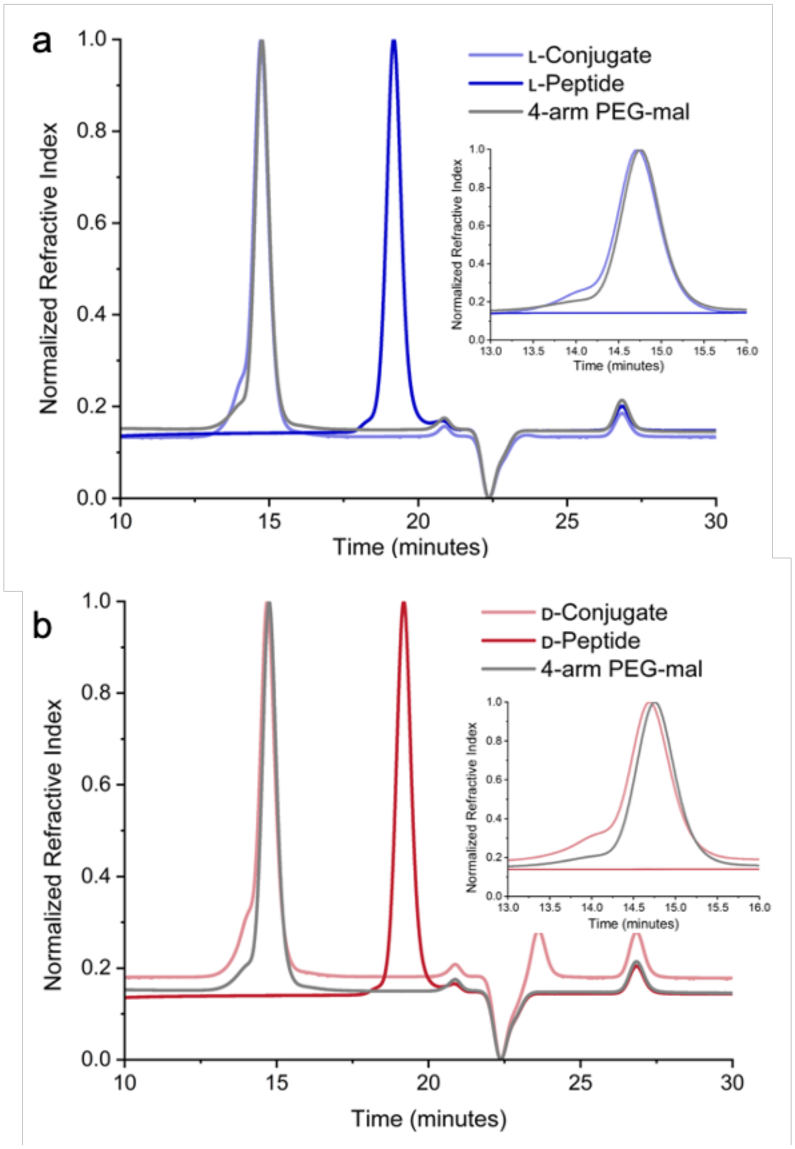

Figure S13. SEC traces of (a) L-KYFILC and (b) D-KYFILC peptides, 4-arm PEG 20k-maleimide, and their conjugates. The disappearance of peptide and polymer peaks in the conjugate traces, and the appearrance of new peak between 14.8 min (inset) suggest the success of conjuagtion.

2.2.5. ^{1}H NMR spectroscopy

We also used ^{1}H NMR spectroscopy to monitor the conjugation of KYFIL peptides with 4-arm PEG-mal. We acquired ^{1}H NMR spectra of the polymers and conjugates and compared the chemical shifts near the thiol and maleimide. In the spectra of 4-arm PEG-mal (Figure S14), the presence of the peak at 6.88 represents the protons on the unsaturated carbons of the 4-arm PEG-male. Upon reaction with the thiol group of the peptides, these unsaturated carbons will be saturated and the peak at 6.88 ppm will disappear. This is exactly what we observed from the NMR spectra of the conjugates (Figure S15 for L-KYFILC conjugates and Figure S16 for D-KYFILC conjugates), which further supported the success of the conjugation. Moreover, we integrated the aromatic protons of phenylalanine and tyrosine (l, m, h and i from 6.75-7.5 ppm in the Figures S15-S16) of the peptide on the conjugates relative to the c' protons at 2.5 ppm on the 4-arm PEG-mal and calculated the percentage of PEG arms functionalized with peptide.

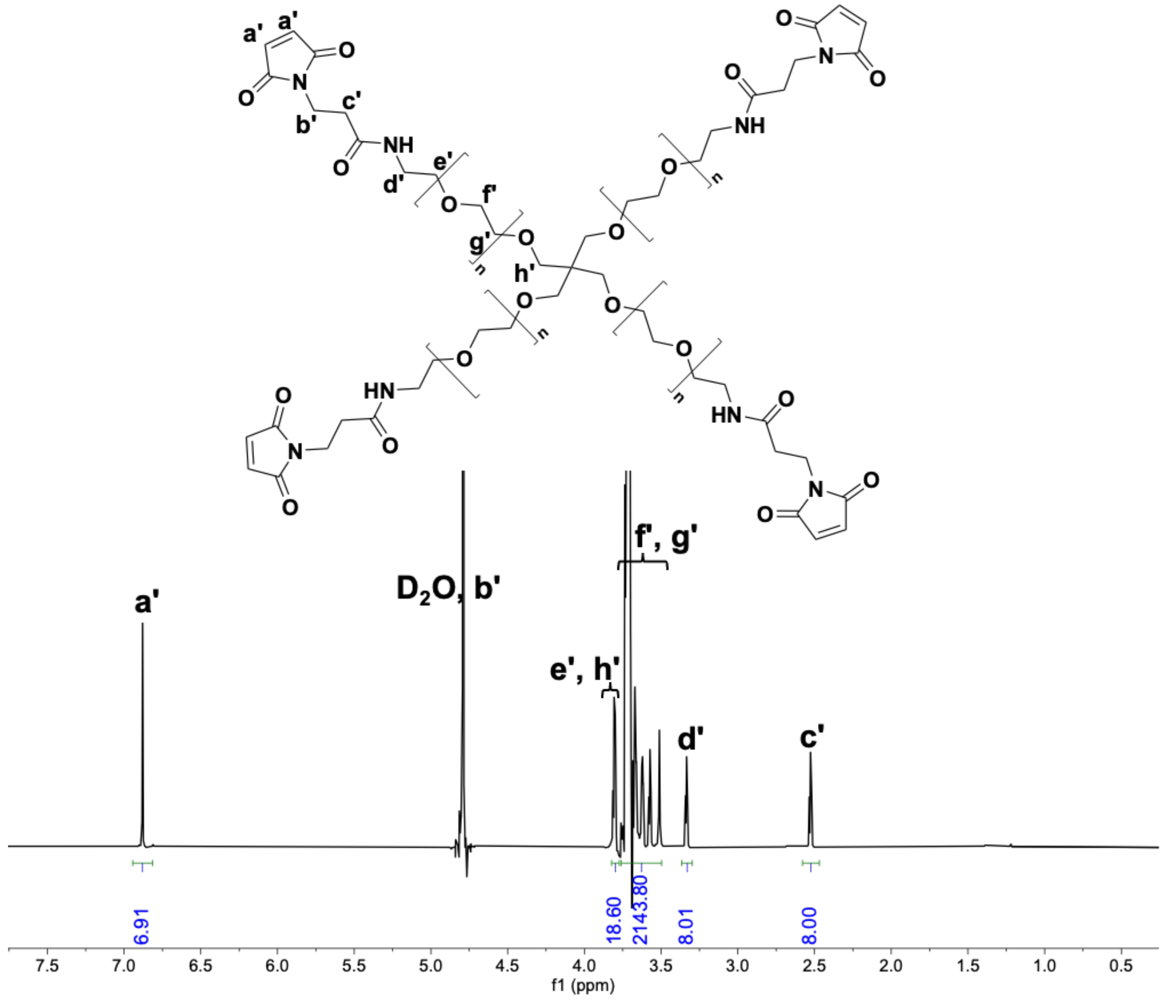

Figure S14: ^{1}H NMR spectrum (800 MHz, D_2O) of 4-arm PEG-maleimide polymer. Protons from the maleimide group appear at 6.88 ppm.

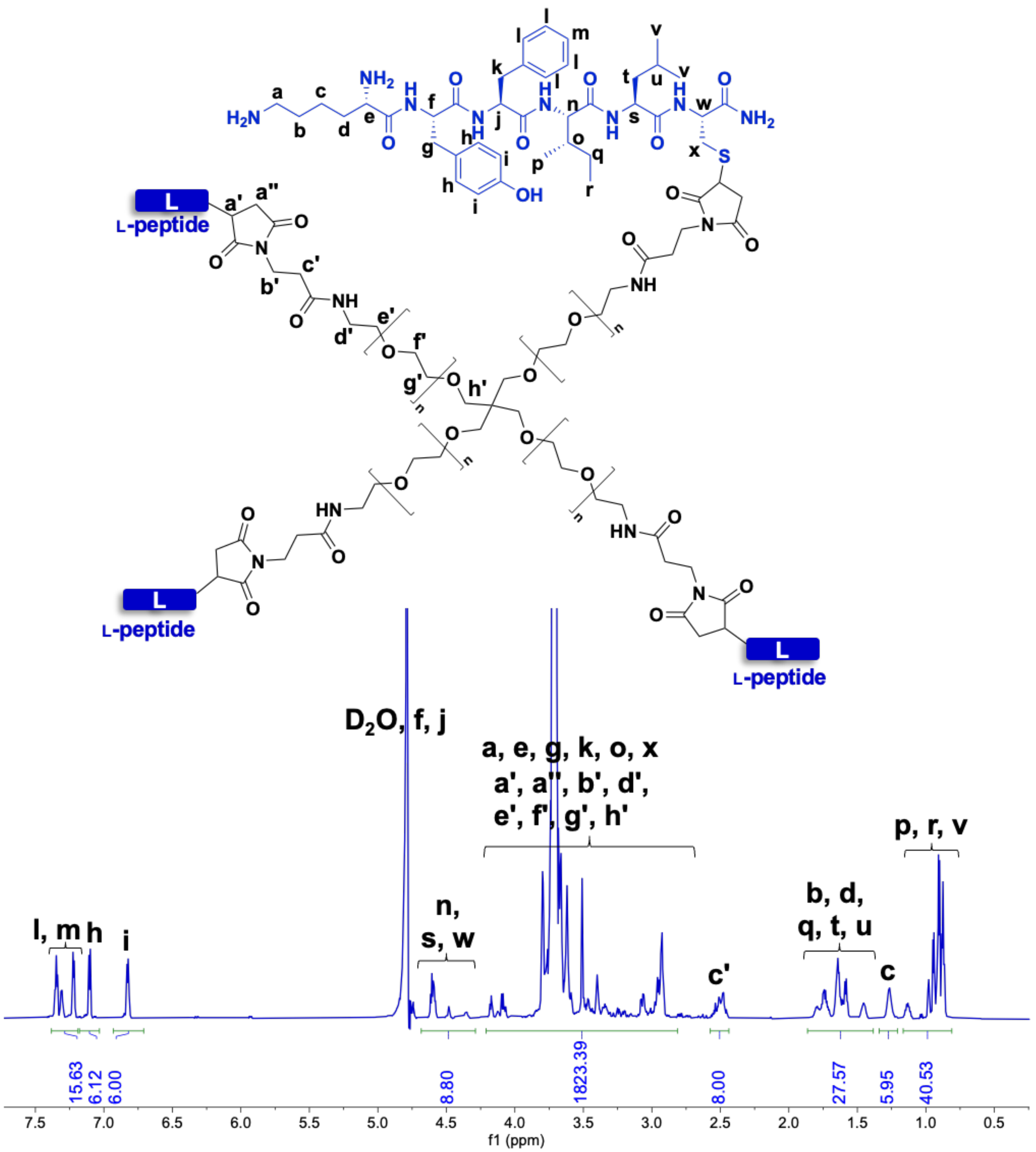

Figure S15: ^{1}H NMR spectra (800 MHz, D_2O) of L-conjugates. The proton resonance from the maleimide group disappears in the conjugate spectra, consistent with conjugation.

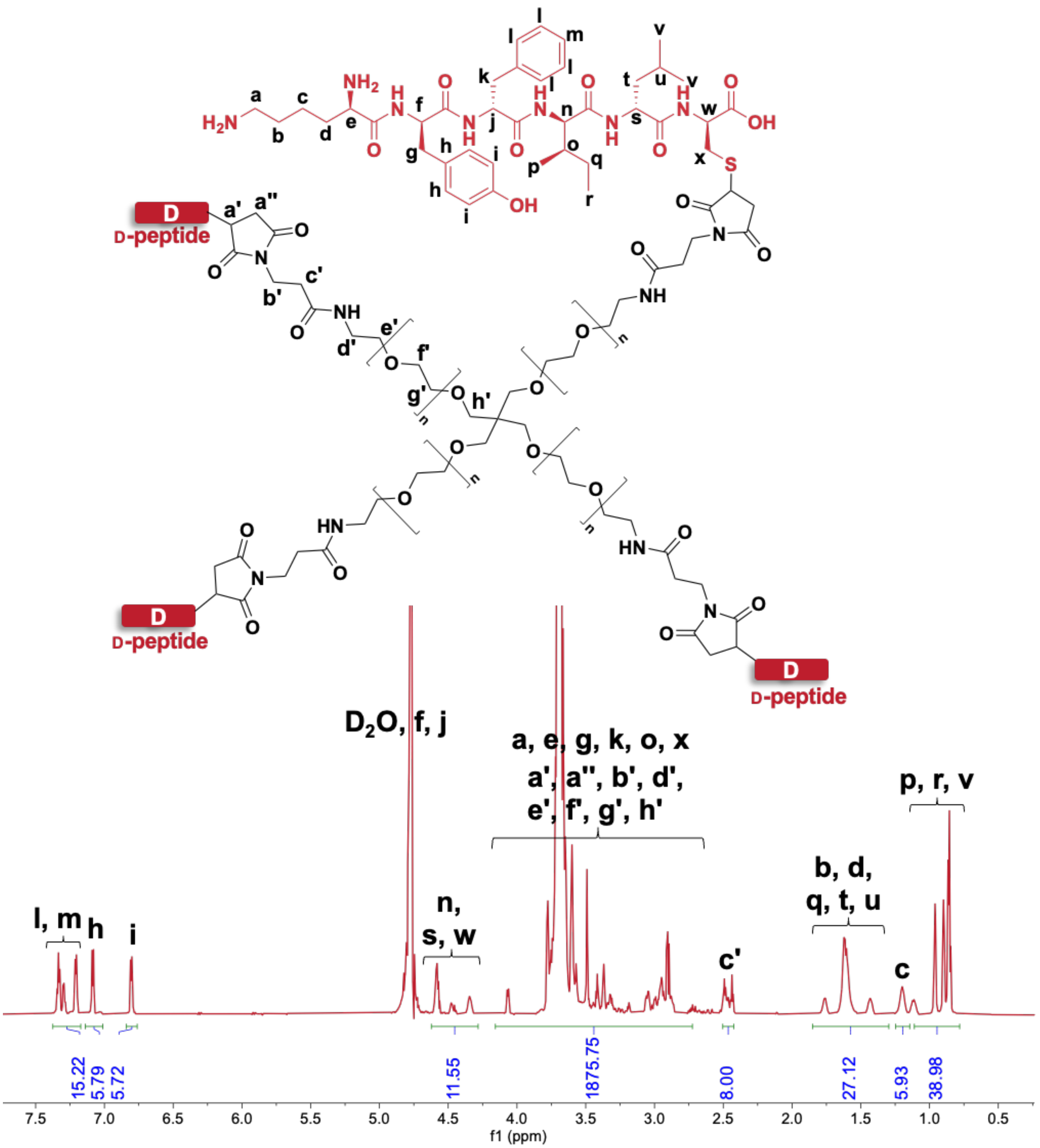

Figure S16: Figure B15: ^{1}H NMR spectra (800 MHz, D_2O) of L- conjugates. The proton resonance from the maleimide group disappears in the conjugate spectra, consistent with conjugation.

2.2.6. Diffusion NMR spectroscopy

Diffusion ordered spectroscopy (DOSY) was used to confirm conjugation of KYFIL peptides to 4-arm PEG-mal by comparing the diffusion coefficients measured for KYFIL-associated resonances in the spectra of KYFIL and the conjugates.[1] By applying spatial gradients in magnetic field strength during an NMR experiment, DOSY detects sample movements over a given amount of time to allow measurement of the diffusion coefficient. In these DOSY experiments, a 90° pulse initially aligns the nuclear spins in the plane perpendicular to the magnet. Then, a gradient pulse is applied with strength G and duration δ that moves magnetically active nuclei in space. After delay time Δ in which the samples diffuse, a second gradient pulse is applied to invert and undo the gradient, followed by acquisition of 1D 1H NMR spectra. The faster the particles diffuse, which is proportional to the gradient strength and inversely proportional to size in solution, the more diffusion occurs during the delay time and the less magnetization will be recovered by the second gradient. Therefore, the gradient pulses encode and decode diffusion information, and the amount of signal recovered as a function of gradient strength can be used to determine the sample's diffusion coefficient. Intensity of the recovered signal vs gradient pulse strength is plotted and fit to this equation: $I_G = I_0 e^{(-\gamma^2 \delta^2 G^2 D_{AB} \Delta')}$, where G is the spatial gradient strength, I_G is the signal intensity at G, I_0 is the signal intensity when G = 2% (the lowest gradient strength, or a gradient that will attenuate just 2% of a signal), δ is the gradient pulse duration, γ is the gyromagnetic ratio, and D_{AB} is the diffusion coefficient of Sample A in Solvent B, and Δ' is the corrected diffusion time.

For data analysis, we imported the 'ser' file in MestReNova 15.0.0. and applied automatic phase and baseline corrections to visualize the attenuation of signal along the 16 gradient strengths, as seen in Figure S17 D-Conjugates. We then performed a DOSY/ROSY transform using the Peak Fit method to obtain the diffusion coefficients of each peak. To calculate a mean diffusion coefficient for a sample, we averaged the diffusion coefficients of each peak associated with the sample, as highlighted in the Figure S18e for the D-Conjugate, excluding artifacts and residual solvent peaks. We also calculated the standard deviations for the associated uncertainty and show these as error bars.

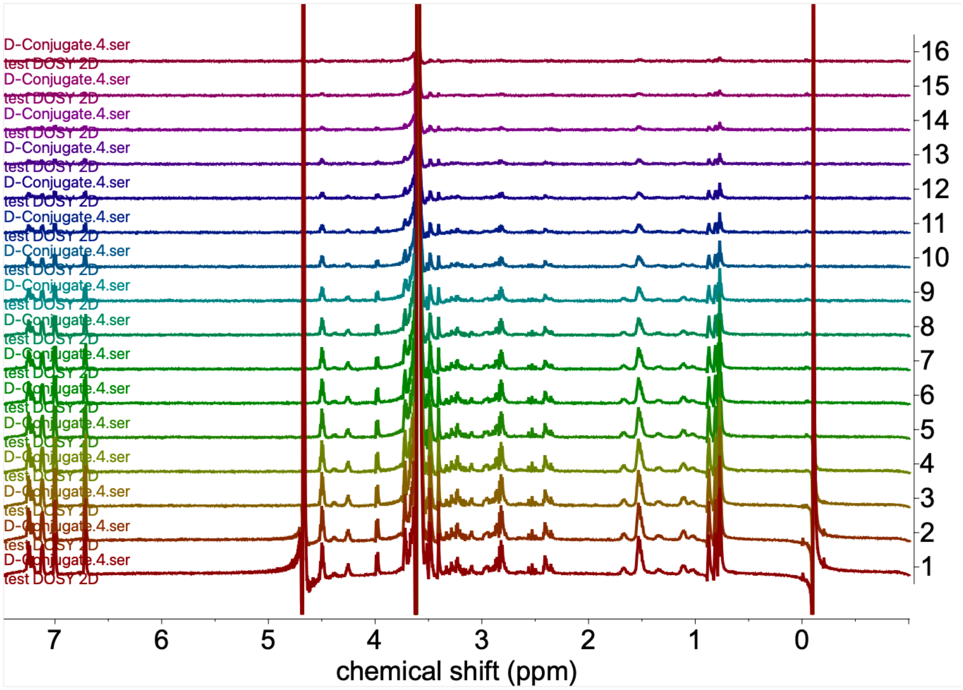

Figure S17: 1D spectra of D-Conjugate at increasing gradient pulse strengths.

Since the delta parameters were fitted using the 4-arm PEG-mal, the smaller peptide samples attenuated quickly, however we verified the peptides were still fit well. For each sample, the Intensity vs Gradient Strength curve is plotted (Figure S18). The circular markers on the plots represent the measured peak intensity at a given gradient strength, while the solid lines represent the curve fit used to calculate the diffusion coefficient of that peak. An ideal Intensity vs Gradient Strength plot looks like the one in Figure S18a, where there are a couple points before substantial decay. In the 2D DOSY contour plots, the diffusion coefficients highlighted in blue are averaged to calculate the overall diffusion coefficients and the standard deviations.

a

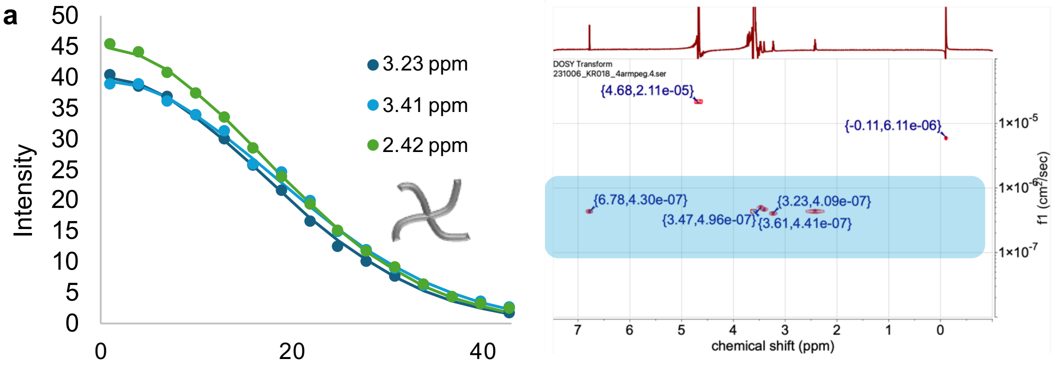

Diffusion Coefficient: $\mathbf{4.11{\times}10^{-7} \pm 2.83{\times}10^{-8}\,\frac{cm^2}{s}}$

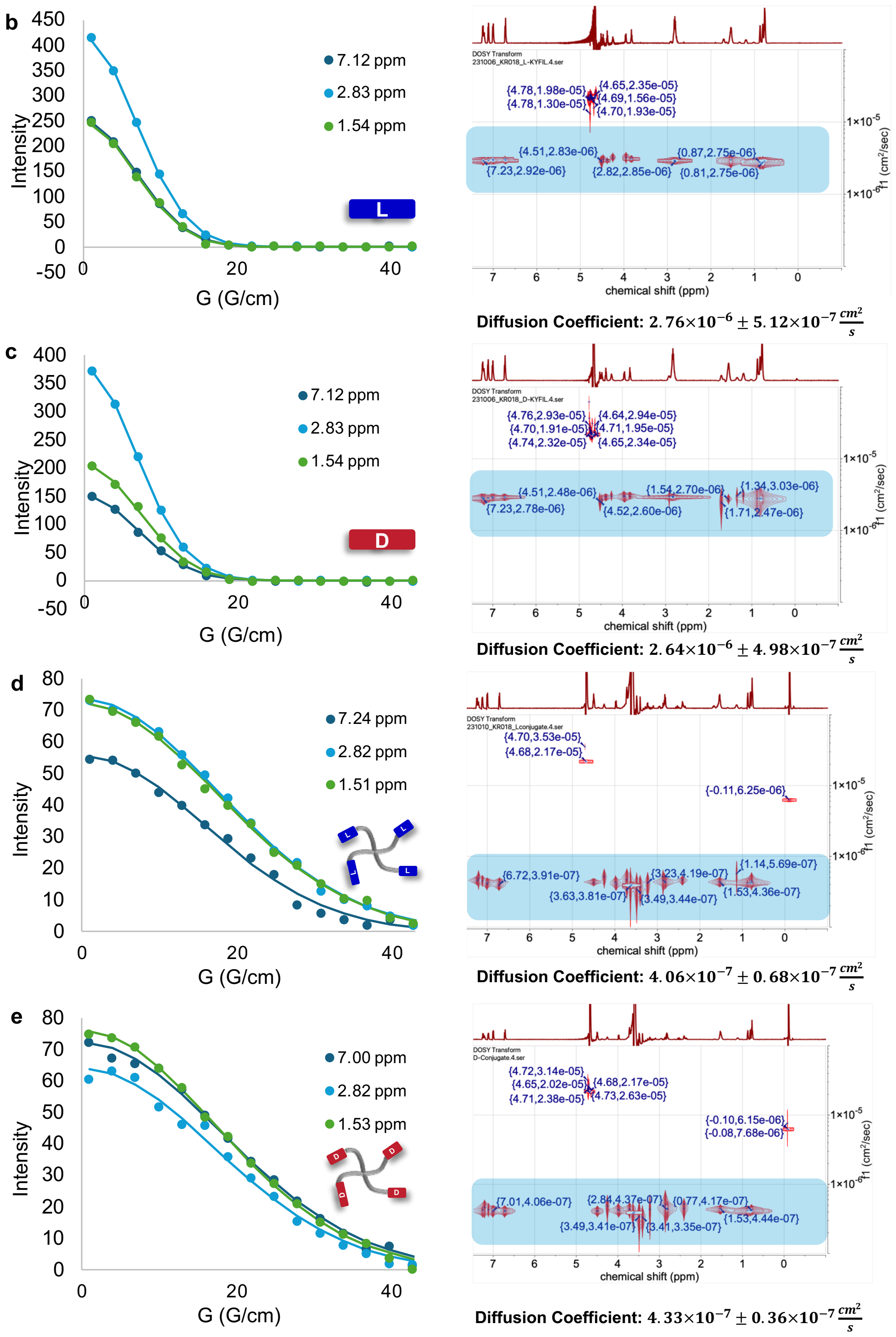

Figure S18: Intensity vs gradient strength curves DOSY counter plots for plots for (a) L-KYFILC, (b) D-KYFILC, (c) L-conjugates, (d) D-KYFILC, and (e) 4-arm PEG-mal.

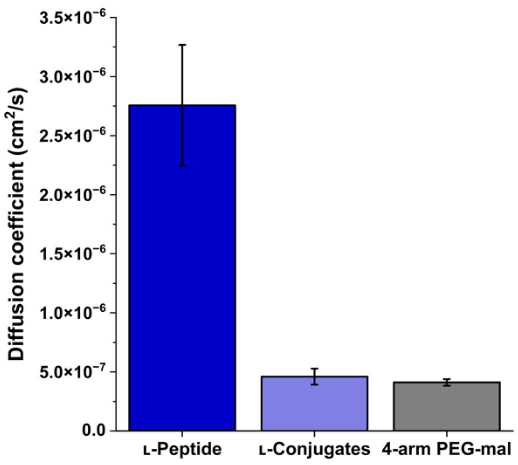

Figure S19: Diffusion coefficient L-KYFILC, 4-arm PEG-mal and L-Conjugates. Diffusivity of the conjugates in D_2O are similar to the diffusivity of polymer and lower than the diffusivity of peptides, indicating peptides' attachment with polymer.

2.2.7. CD spectroscopy

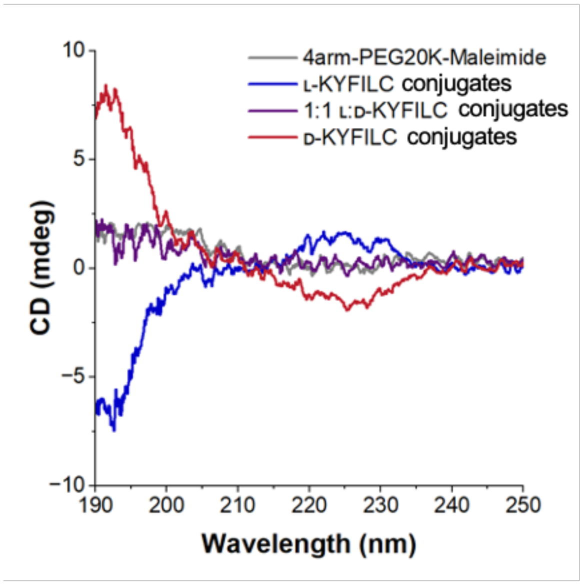

Figure S20: Circular dichroism spectra of L-,1:1 L:D-, D-conjugates and 4-arm PEG-mal at 0.59% (w/v) in water.

2.3. Hydrogel formation

2.3.1. Images of conjugates in PBS

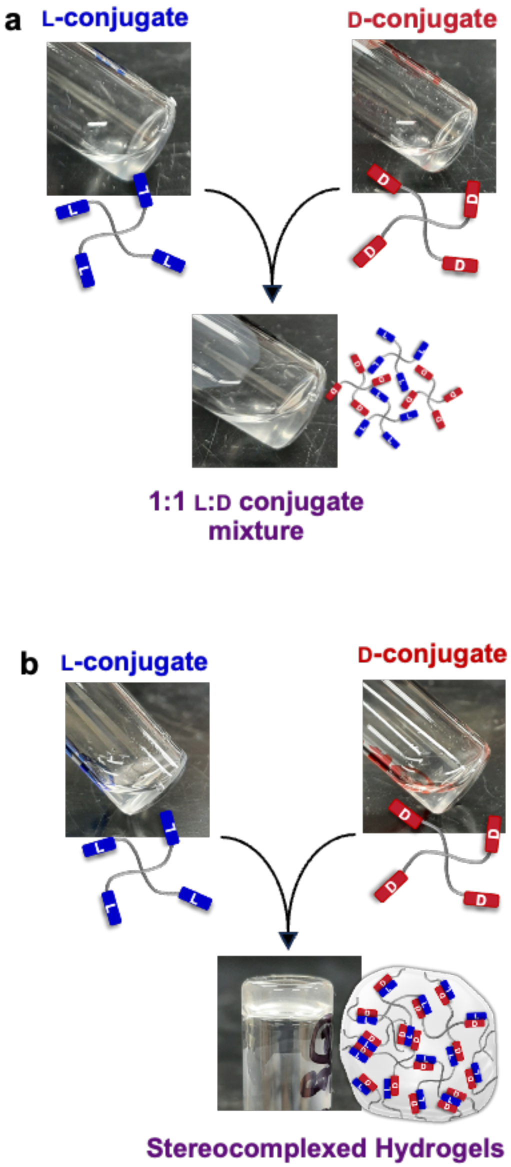

Figure S21: Images of L-,1:1 L:D- and D-conjugates at (a) 5% (w/v) and (b) 10% (w/v).

2.3.2. IR spectroscopy

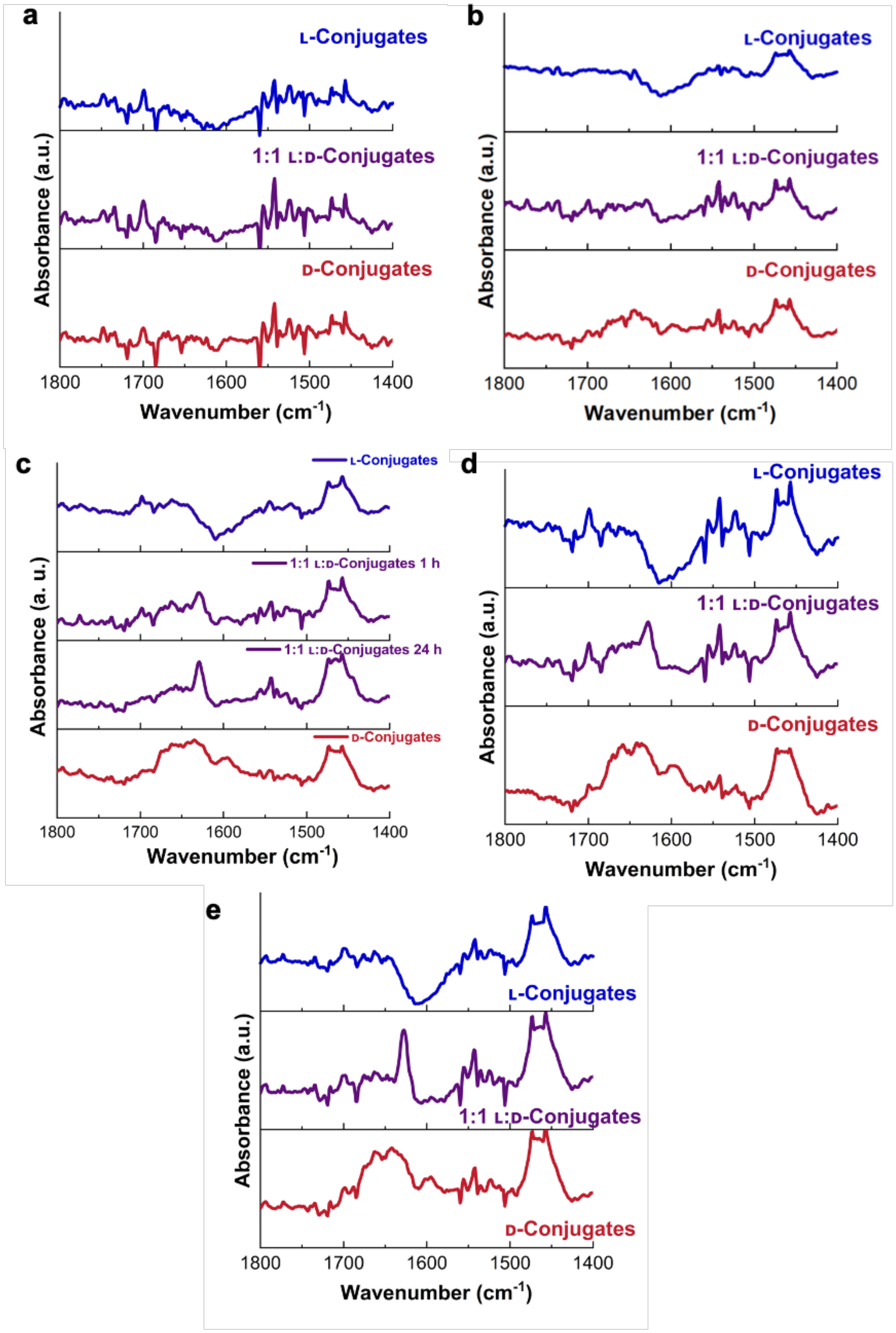

Figure S22: FTIR spectra of L-,1:1 L:D- and D-conjugates at (a) 3% (w/v), (b) 5% (w/v), (c) 7.5%, (d) 10% (w/v), and (e) 15% (w/v). Individual L- and D-conjugates do not form β-sheet at any of the

tested concentrations. The 1:1 mixture of L- and D-conjugates show absorbances at 1630 cm^{-1} at 7.5% (w/v) and higher concentrations, indicative of β-sheet formation.

2.4. Rheology

In this section, we provide our full set of rheology data. Using amplitude sweeps, we identified the linear viscoelastic strain regime from which we selected strain conditions for subsequent frequency sweeps.

2.4.1. Amplitude sweeps at 5% (w/v)

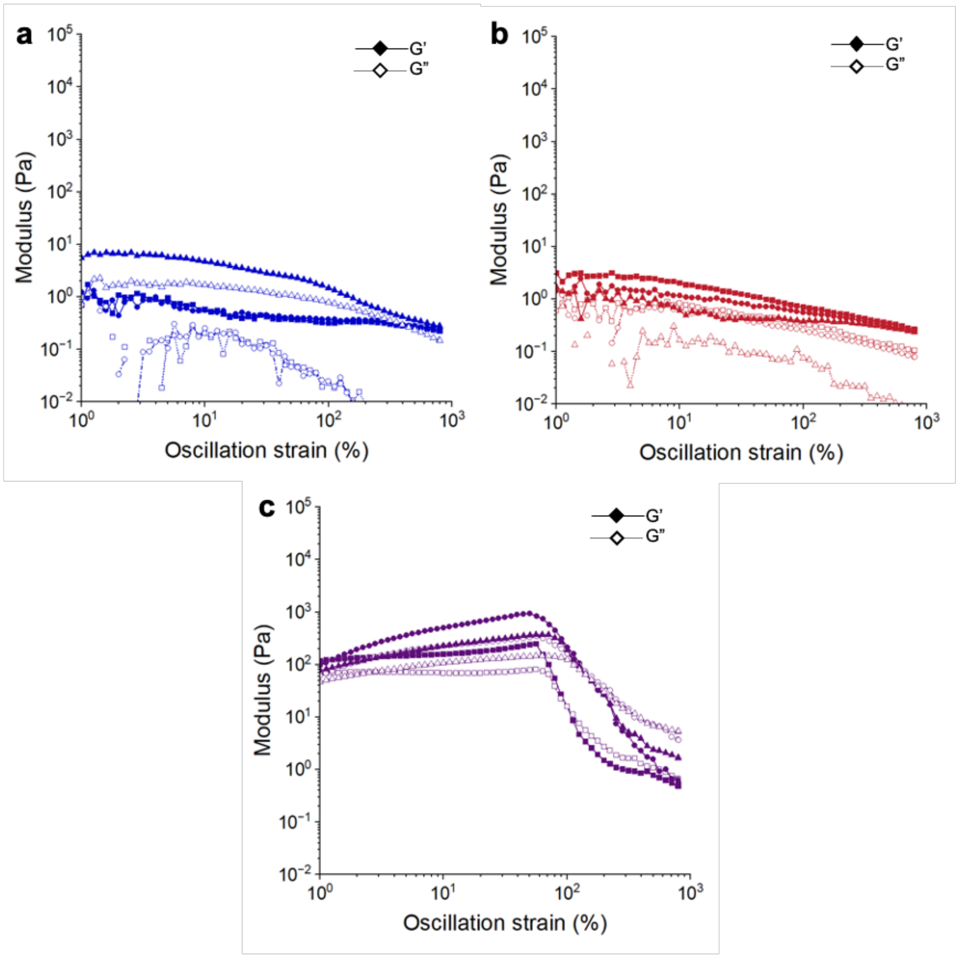

Figure S23. Amplitude sweeps of KYFIL conjugates at 5% (w/v) in PBS. (a) L-conjugates, (b) D-conjugates, and (c) 1:1 L:D-conjugates were subjected to oscillatory shear using a 8 mm diameter parallel plate geometry with a 500 μm gap height at 1 rad/s and 25°C, ramping logarithmically from 1% to 800% strain. Solid symbols represent storage moduli (G'); open symbols represent loss moduli (G"). These triplicate runs from three independently prepared samples were used.

The artifacts in the L- and D-conjugate samples are attributed to the use of a parallel plate geometry for these liquid samples.

2.4.2. Amplitude sweeps at 7.5% (w/v)

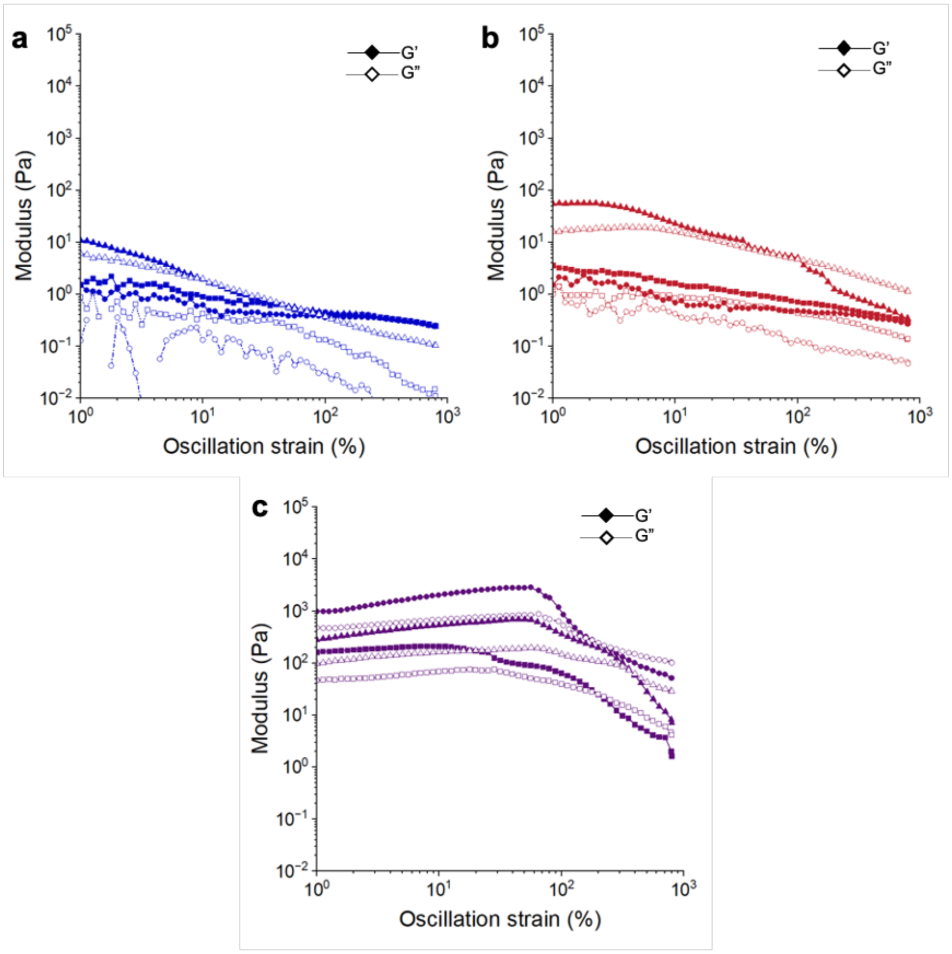

Figure S24. Amplitude sweeps of KYFIL conjugates at 7.5% (w/v) in PBS. (a) L-conjugates, (b) D-conjugates, and (c) 1:1 L:D-conjugates were subjected to oscillatory shear using an 8 mm diameter parallel plate geometry with a 500 μm gap height at 1 rad/s and 25°C, ramping logarithmically from 1% to 800% strain. Solid symbols represent storage moduli (G'); open symbols represent loss moduli (G"). These measurements were run in triplicate on three independently prepared samples. The artifacts in the L- and D-conjugate samples are attributed to the use of a parallel plate geometry for these liquid samples.

2.4.3. Amplitude sweeps at 10% (w/v)

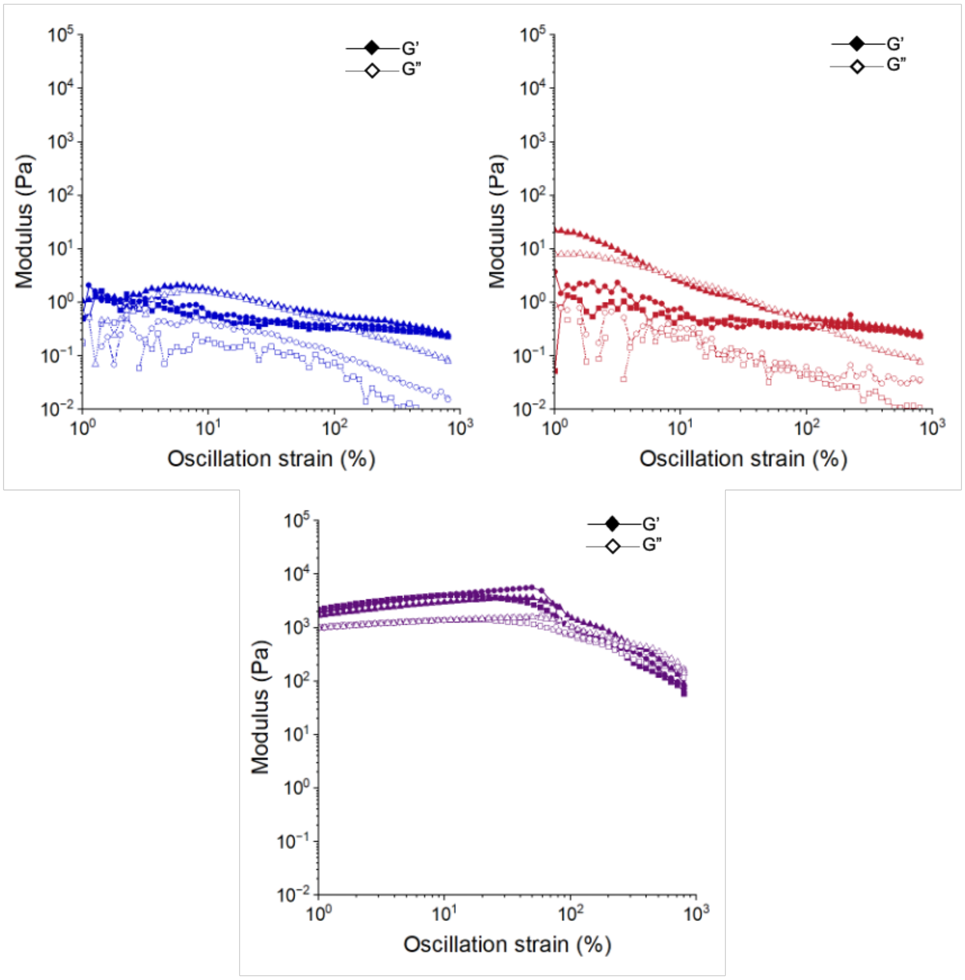

Figure S25. Amplitude sweeps of KYFIL conjugates at 10% (w/v) in PBS. (a) L-conjugates, (b) D-conjugates, and (c) 1:1 L:D-conjugates were subjected to oscillatory shear using a 8 mm diameter parallel plate geometry with a 500 μm gap height at 1 rad/s and 25°C, ramping logarithmically from 1% to 800% strain. Solid symbols represent storage moduli (G'); open symbols represent loss moduli (G"). These measurements were run in triplicate on three independently prepared samples. The artifacts in the L- and D-conjugate samples are attributed to the use of a parallel plate geometry for these liquid samples.

2.4.4. Frequency sweeps at 5% (w/v)

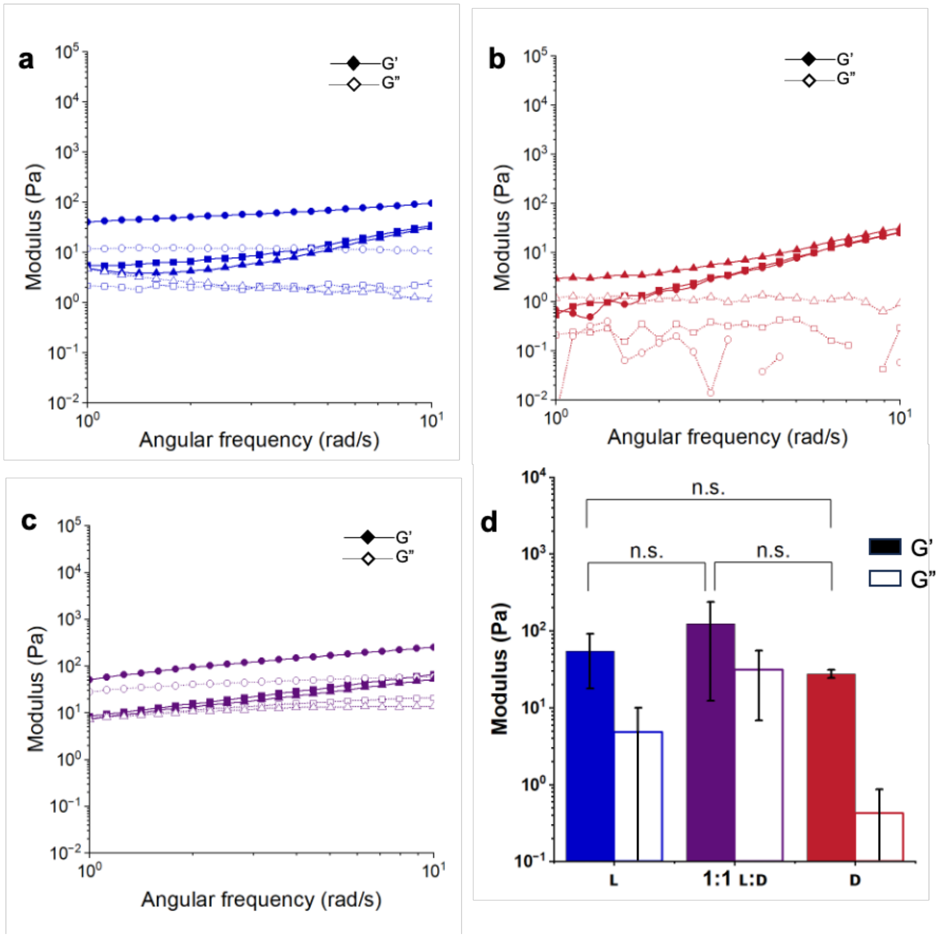

Figure S26. Frequency sweeps of KYFIL conjugates at 5% (w/v) in PBS. Oscillatory shear tests on (a) L-conjugates, (b) D-conjugates, and (c) 1:1 L:D-conjugates and (d) a comparison of the average storage and loss moduli. Three runs on independently prepared samples, using an 8 mm diameter parallel plate geometry with a 500 μm gap height at 5% strain and 25°C, ramping angular frequency logarithmically from 1 to 10 rad/s. Solid symbols represent storage moduli (G'); open symbols represent loss moduli (G"). L- and D-conjugates runs contain artifacts attributed to the use of the parallel plate geometry for these liquid samples. We suspect that the parallel plate geometry, selected for the stereocomplexed hydrogel samples with solid-like character, led to higher variability and artifacts in the liquid-like solutions.

2.4.5. Frequency sweeps at 7.5% (w/v)

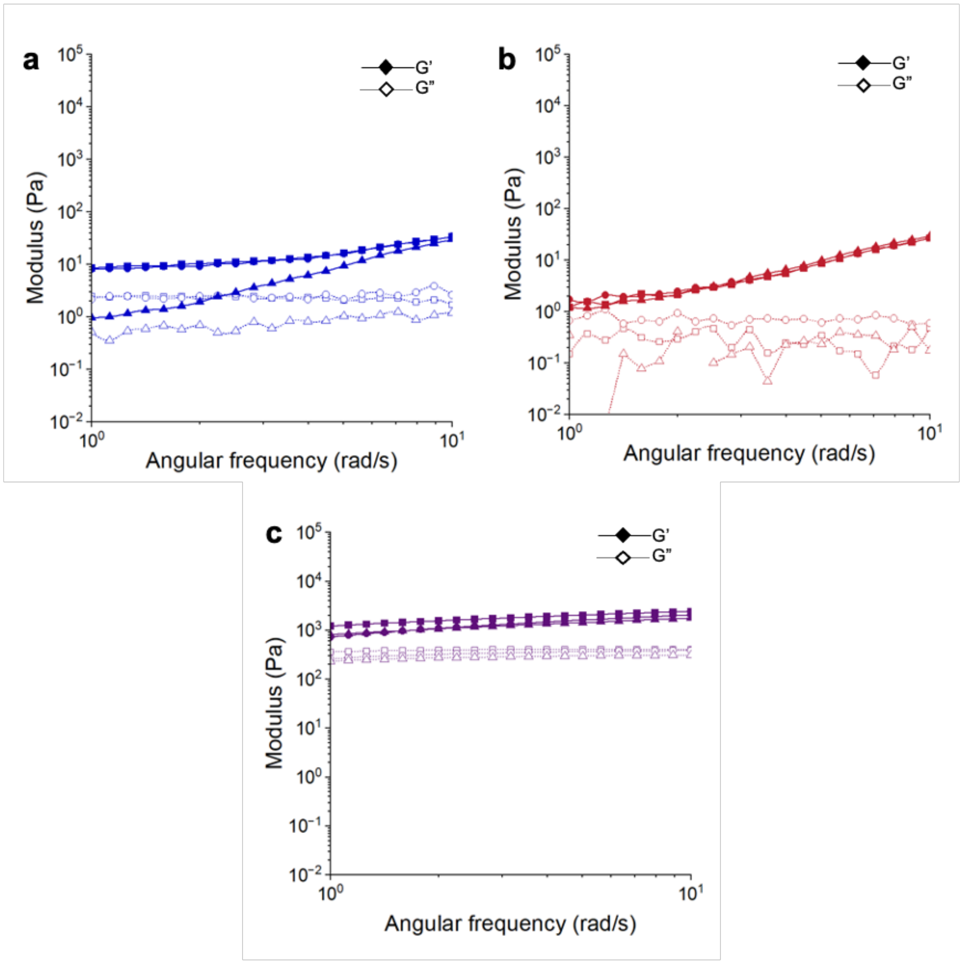

Figure S27. Frequency sweeps of KYFIL conjugates at 7.5% (w/v) in PBS. Oscillatory shear tests on (a) L-conjugates, (b) D-conjugates, and (c) 1:1 L:D-conjugates, three runs from independently prepared samples, using a 8 mm diameter parallel plate geometry with a 500 μm gap height at 5% strain and 25°C, ramping angular frequency logarithmically from 1 to 10 rad/s. Solid symbols represent storage moduli (G'); open symbols represent loss moduli (G"). L- and D-conjugates runs contain artifacts attributed to the use of the parallel plate geometry for these liquid samples.

2.4.6. Frequency sweeps at 10% (w/v)

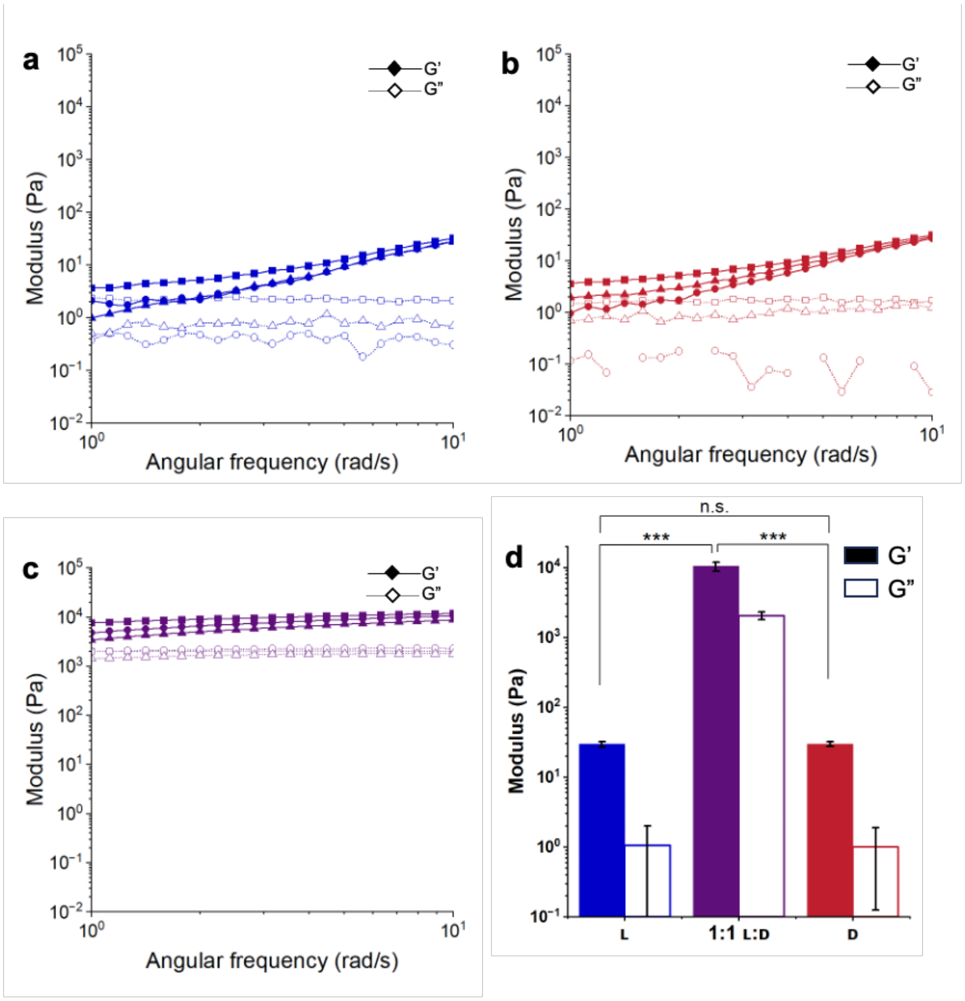

Figure S28. Frequency sweeps of KYFIL conjugates at 10% (w/v) in PBS. Oscillatory shear tests on (a) L-conjugates, (b) D-conjugates, and (c) 1:1 L:D-conjugates and (d) a comparison of the average storage and loss moduli. Three runs on independently prepared samples, using a 8 mm diameter parallel plate geometry with a 500 μm gap height at 5% strain and 25°C, ramping angular frequency logarithmically from 1 to 10 rad/s. Solid symbols represent storage moduli (G'); open symbols represent loss moduli (G"). L- and D-conjugates runs contain artifacts attributed to the use of the parallel plate geometry for these liquid samples. The error bars represent standard deviation, with n = 3 samples.

2.4.7. Stress recovery of hydrogels at 7.5% (w/v)

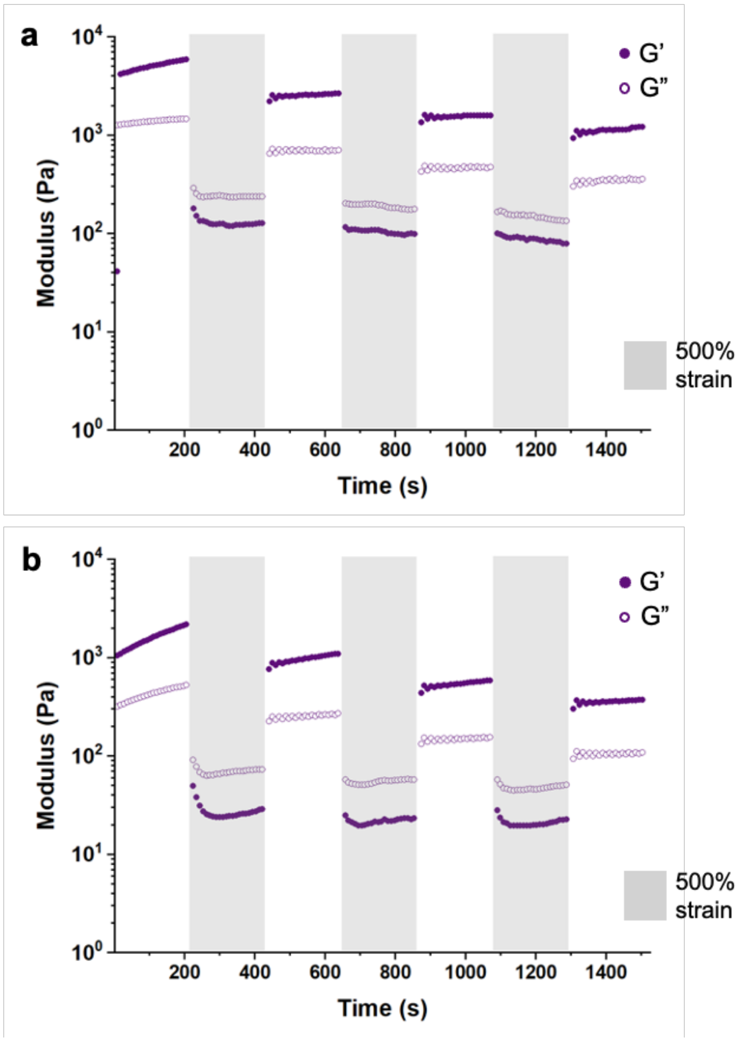

Figure S29. Dynamic behavior of stereocomplexed hydrogels (on two independently prepared samples) at 7.5% (w/v) in 1X PBS. Hydrogels were subjected to three cyclic applications of 500% strain for 200 s (grey area), followed by 5% strain for 200 s. Hydrogels recover ~10-50% after the first cycle, and for the remaining cycles the recoveries were ~50-60%.

2.4.8. Stress recovery of hydrogels at 10% (w/v)

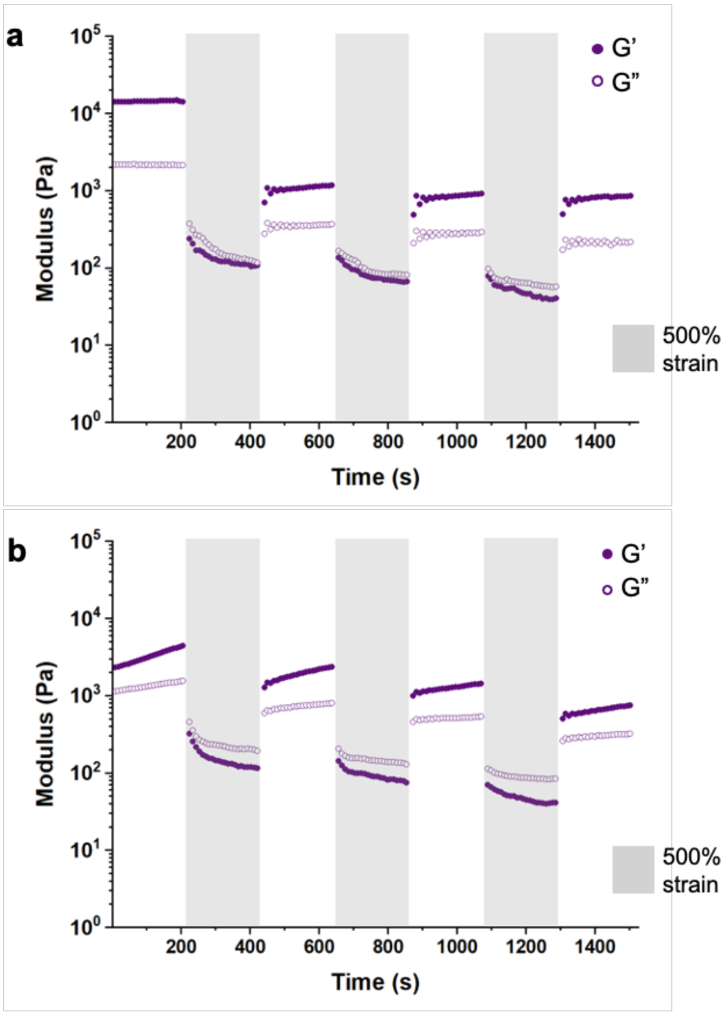

Figure S30. Dynamic behavior of stereocomplexed hydrogels (on two independently prepared samples) at 10% (w/v) in 1X PBS (a-b). Hydrogels were subjected to three cyclic applications of 500% strain for 200 s (grey area), followed by 5% strain for 200 s. Hydrogels recover ~10-50% after the first cycle, and for the remaining cycles the recoveries were ~60-70%.

2.4.9. Stress recovery experiments on hydrogels over longer times

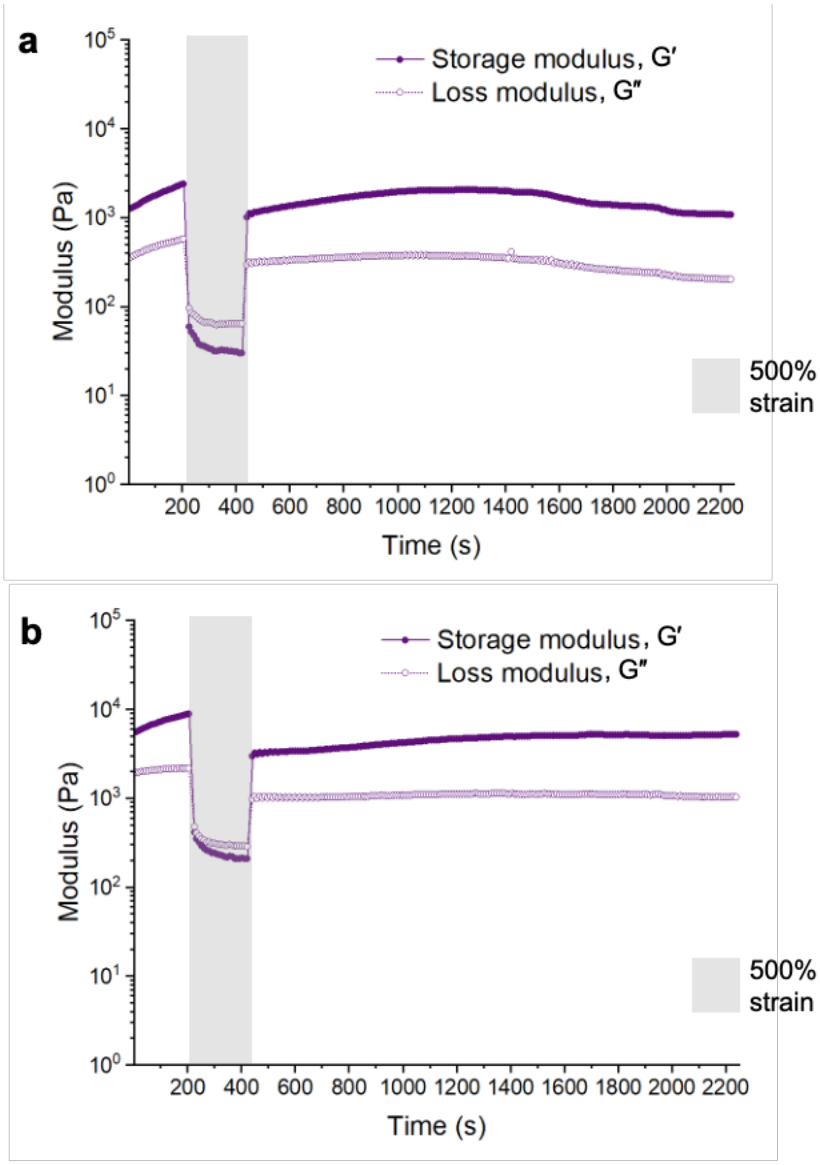

Figure S31. Stress recovery of hydrogels in 1X PBS at (a) 7.5% (w/v) and (b) 10% (w/v) with longer recovery time. Hydrogels were subjected to 500% strain for 200 s (grey area), followed by 5% strain for 30 min. Hydrogels modulus plateaued 15-20 mins following removal of high strain and the 7.5% (w/v) and 10% (w/v) formulations recovered ~50% and 60% of their original modulus, respectively.

2.5. X-ray diffraction patterns

X-ray diffraction (XRD) was conducted to assess the impact of the polymer on peptide crystallinity. We prepared the 4-arm PEG20k-maleimide, L-conjugates, D- conjugates, and the 1:1 L:D conjugate mixture by dissolving the 10% (w/v) sample in 1X PBS, incubating samples at room temperature for 30 min and 24 h, followed by lyophilization and diffraction. XRD patterns reveal prominent peaks at 2θ = 19º and 23º in the 4-arm PEG-mal, which persists in the conjugated samples at both time points (Figure S32). PEG is well hydrated in solution and not likely to crystallize, so we attribute these peaks to drying-induced crystallization.[9] Since the PEG-related signals dominate intensity in these lyophilized peaks, the peaks related to peptide crystallization were less prominent. We did not observe peaks at 2θ = 8.6 and 19.4º, previously seen in unconjugated KYFIL stereocomplexes.[2] However, a subtle yet distinct increase in peak intensity appears at 2θ = 27º in the stereocomplex relative to those in the patterns of the individual conjugates, which suggests the stereocomplexed conjugates interact with salt in a different manner. We found in the literature that when stereocomplexed forming polymers are conjugated with PEG, their associated crystalline peaks become less prominent in their XRD patterns. For example, for the stereocomplexed polylactide-PEG conjugates with lower relative PEG content, a small peak appears at 2θ = 12 º and 21 º corresponding to the stereocomplex crystal.[10] Between drying-induced crystallization and the high PEG content in this study, PEG crystallization makes it difficult to conclusively elucidate the stereocomplexation-driven crystallinity changes in these samples. However the reproducible, subtle changes we observe in two independently prepared samples, suggest stereocomplexation-induced crystallinity changes are occurring, a notion that is further supported by prominent peaks characteristic of β-sheets in the infrared spectra.

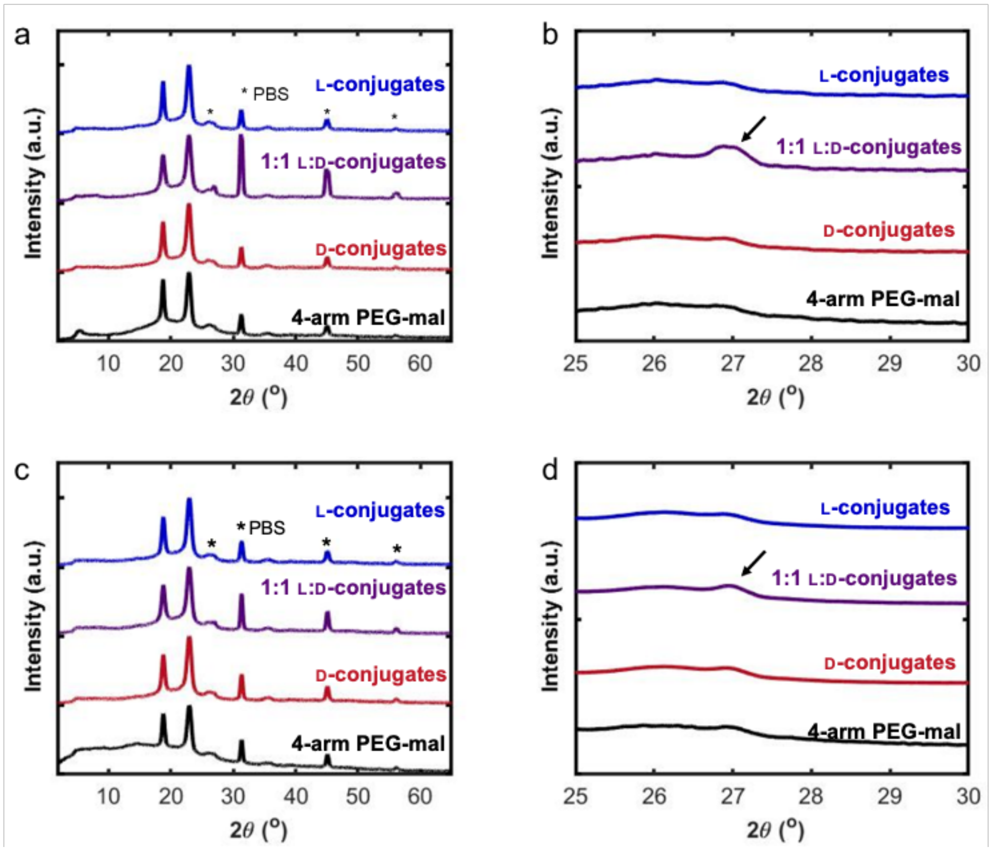

Figure S32: X-ray diffraction patterns of samples after 30 min (a,b) and after 24 h (c,d). PEG crystallization intensity dominates in all four samples, obscuring KYFILC crystal features. PBS salt peaks appear at 2θ = 27º, 31.4º, 45º, and 56º. Subtle feature in the 1:1 L:D-KYFILC conjugate appears at 2θ = 27º for both time points.

2.6. Proteolytic stability measurement

2.6.1. HPLC chromatograms of controls

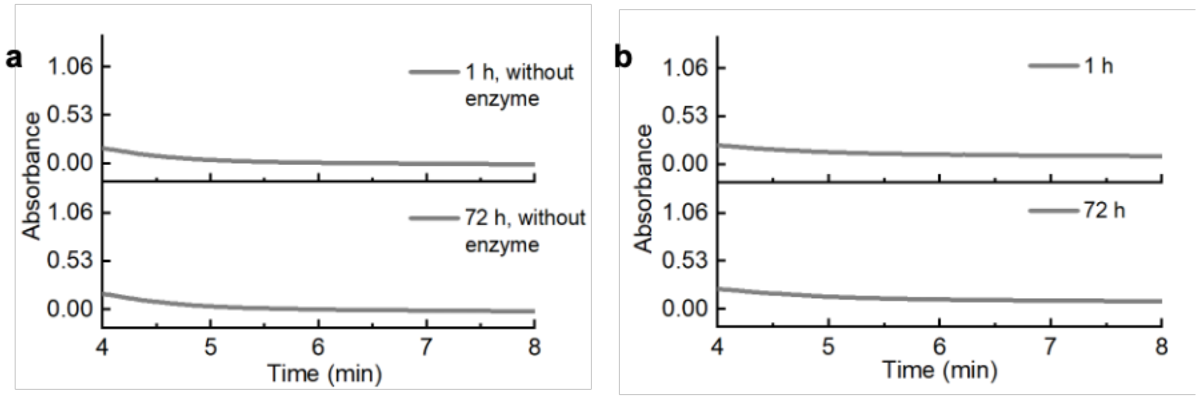

Figure S33. HPLC chromatograms of 1X PBS with 0.1 mg/mL Preteinase K. We observed no absorbance at 214 nm from 4-8 min.

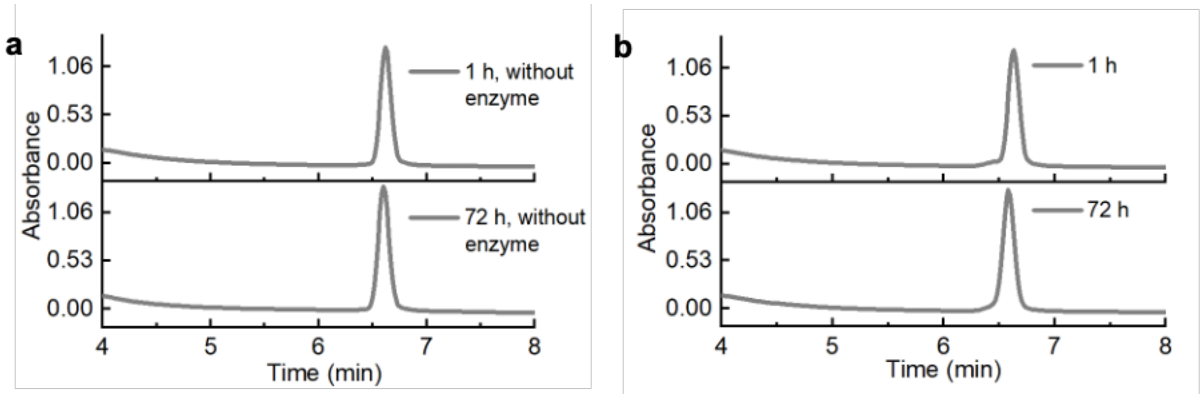

Figure S34. Control stability measurements of 4-arm PEG-mal in (a) absence and (b) presence of Proteinase K enzyme. No degradation was observed from the chromatograms of PEG, allowing us to focus on the degradation of the peptides on the conjugates.

2.6.2. HPLC chromatograms of L-conjugates

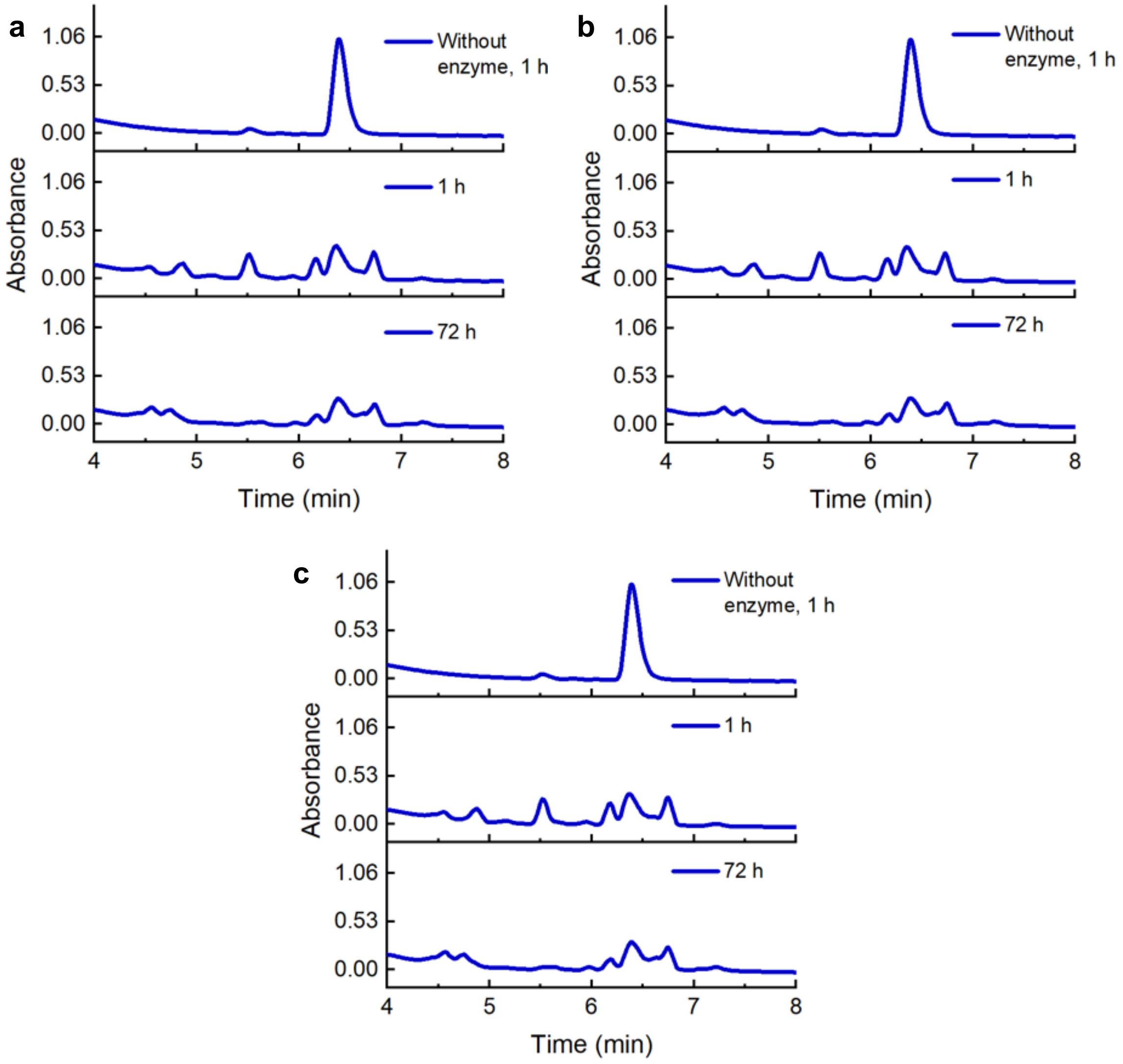

Figure S35. Proteolytic stability of L-conjugates. (a-c) HPLC traces acquired of 3 different samples after incubation of the materials without enzyme for 1 h and with Proteinase K (0.1 mg/mL) for 1 and 72 h. The appearance of new peaks after incubation with enzymes indicates degradation.

2.6.3. HPLC chromatograms of D-conjugates

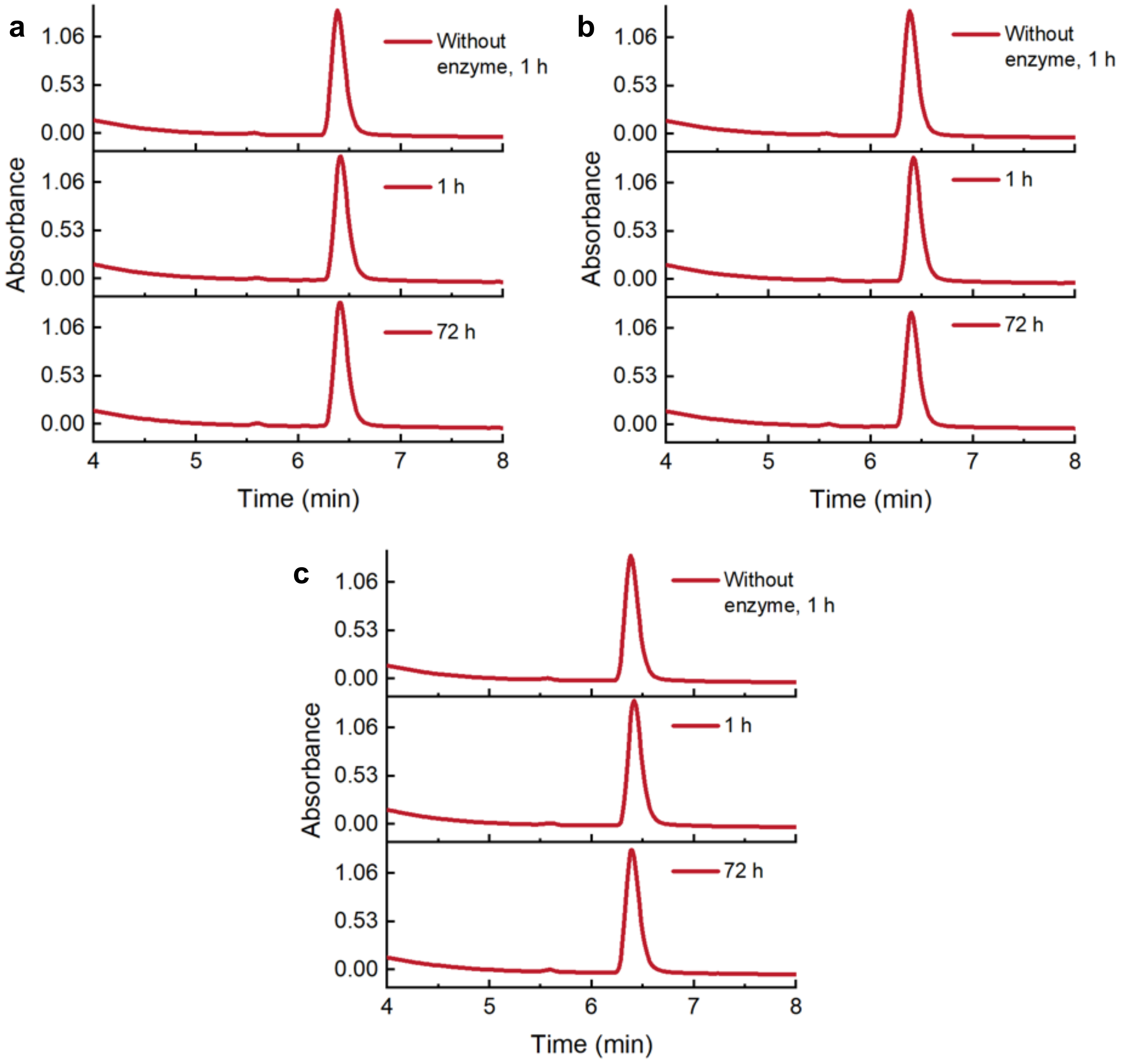

Figure S36: Proteolytic stability of D-conjugates. (a-c) HPLC traces acquired of 3 different samples after incubation of the materials without enzyme for 1 h and with Proteinase K (0.1 mg/mL) for 1 and 72 h. We observe no degradation of D-conjugates.

2.6.4. HPLC chromatograms of 1:1 L:D-conjugates

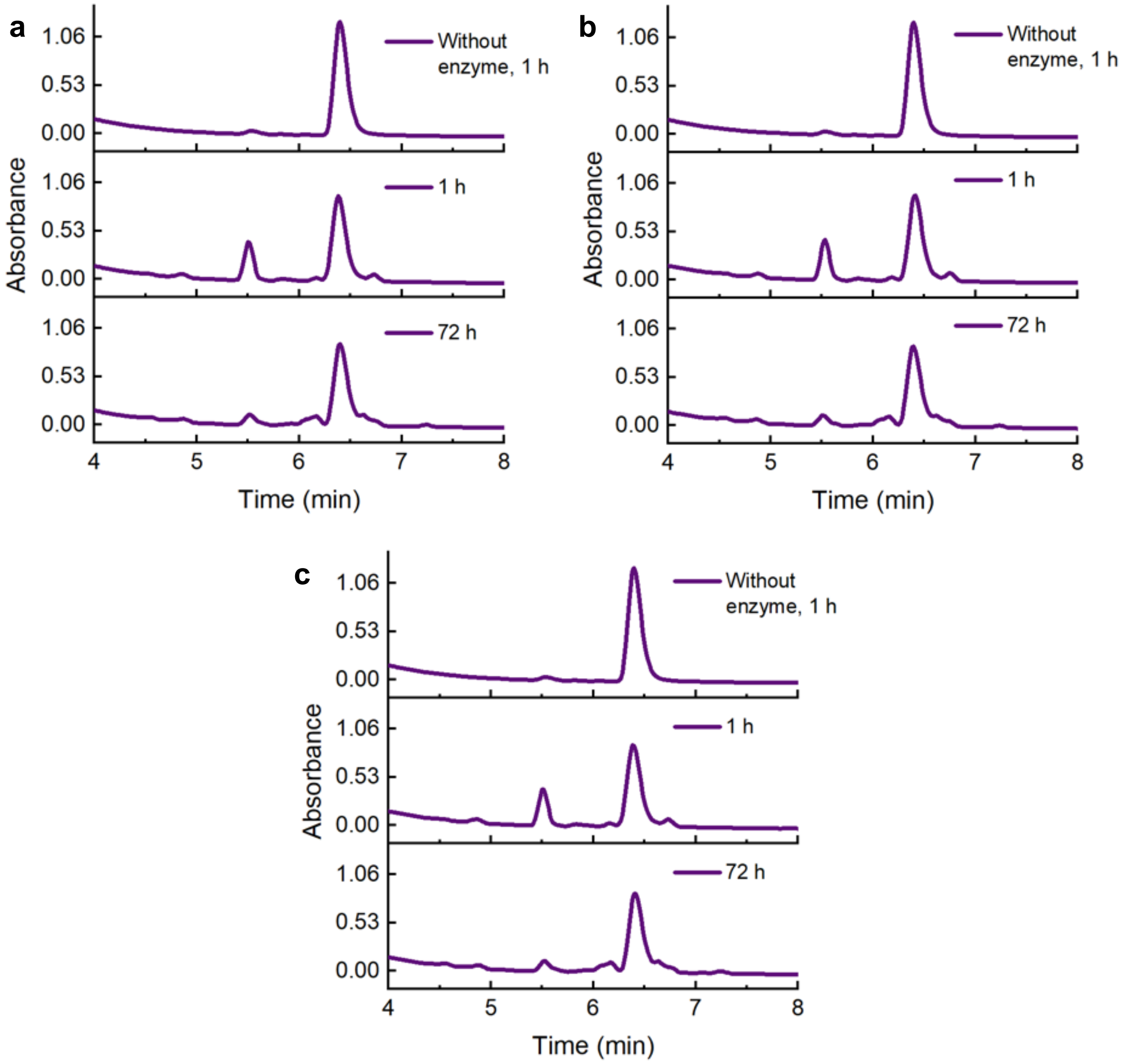

Figure S37: Proteolytic stability of 1:1 L:D-hydrogels. (a-c) HPLC traces acquired of 3 different samples after incubation of the materials without enzyme for 1 h and with Proteinase K (0.1 mg/mL) for 1 and 72 h. More than 75% of the conjugates were intact after 1 h, indicating stereocomplexation shields L-peptides from proteolytic degradation.

3. References

1 P. Groves, *Polym. Chem.*, 2017, **8**, 6700–6708.

2 I. J. Duti, J. R. Florian, A. R. Kittel, C. D. Amelung, V. P. Gray, K. J. Lampe and R. A. Letteri, *J. Am. Chem. Soc.*, 2023, **145**, 18468–18476.

3 A. Caballero-Herrera, K. Nordstrand, K. D. Berndt and L. Nilsson, *Biophys. J.*, 2005, **89**, 842–857.

4 I. Jahan and S. M. Nayeem, *ACS Omega*, 2018, **3**, 11727–11741.

5 N. Steinke, R. J. Gillams, L. C. Pardo, C. D. Lorenz and S. E. McLain, *Phys. Chem. Chem. Phys.*, 2016, **18**, 3862–3870.

6 M. C. Stumpe and H. Grubmüller, *J. Am. Chem. Soc.*, 2007, **129**, 16126–16131.

7 L. B. Sagle, Y. Zhang, V. A. Litosh, X. Chen, Y. Cho and P. S. Cremer, *J. Am. Chem. Soc.*, 2009, **131**, 9304–9310.

8 A. N. Elder, N. M. Dangelo, S. C. Kim and N. R. Washburn, *Biomacromolecules*, 2011, **12**, 2610–2616.

9 D. O. Corrigan, A. M. Healy and O. I. Corrigan, *Int. J. Pharm.*, 2002, **235**, 193–205.

10 C. Wang, N. Feng, F. Chang, J. Wang, B. Yuan, Y. Cheng, H. Liu, J. Yu, J. Zou, J. Ding and X. Chen, *Adv. Healthc. Mater.*, 2019, **8**, 1900312.
